# Dual prognostic role of 2-oxoglutarate-dependent oxygenases in ten cancer types: implications for cell cycle regulation and cell adhesion maintenance

**DOI:** 10.1186/s40880-019-0369-5

**Published:** 2019-04-29

**Authors:** Wai Hoong Chang, Donall Forde, Alvina G. Lai

**Affiliations:** Nuffield Department of Medicine, University of Oxford, Old Road Campus, Oxford, OX37FZ UK

**Keywords:** Oxygen-sensing gene, 2-Oxoglutarate-dependent oxygenase, Pan-cancer, Prognosis, Hypoxia, *KDM8*, *HNF4A*

## Abstract

**Background:**

Tumor hypoxia is associated with metastasis and resistance to chemotherapy and radiotherapy. Genes involved in oxygen-sensing are clinically relevant and have significant implications for prognosis. In this study, we examined the pan-cancer prognostic significance of oxygen-sensing genes from the 2-oxoglutarate-dependent oxygenase family.

**Methods:**

A multi-cohort, retrospective study of transcriptional profiles of 20,752 samples of 25 types of cancer was performed to identify pan-cancer prognostic signatures of 2-oxoglutarate-dependent oxygenase gene family (a family of oxygen-dependent enzymes consisting of 61 genes). We defined minimal prognostic gene sets using three independent pancreatic cancer cohorts (n = 681). We identified two signatures, each consisting of 5 genes. The ability of the signatures in predicting survival was tested using Cox regression and receiver operating characteristic (ROC) curve analyses.

**Results:**

Signature 1 (*KDM8, KDM6B, P4HTM, ALKBH4, ALKBH7*) and signature 2 (*KDM3A, P4HA1, ASPH, PLOD1, PLOD2*) were associated with good and poor prognosis. Signature 1 was prognostic in 8 cohorts representing 6 cancer types (n = 2627): bladder urothelial carcinoma (*P* = 0.039), renal papillary cell carcinoma (*P* = 0.013), liver cancer (*P* = 0.033 and *P* = 0.025), lung adenocarcinoma (*P* = 0.014), pancreatic adenocarcinoma (*P* < 0.001 and *P* = 0.040), and uterine corpus endometrial carcinoma (*P* < 0.001). Signature 2 was prognostic in 12 cohorts representing 9 cancer types (n = 4134): bladder urothelial carcinoma (*P* = 0.039), cervical squamous cell carcinoma and endocervical adenocarcinoma (*P* = 0.035), head and neck squamous cell carcinoma (*P* = 0.038), renal clear cell carcinoma (*P* = 0.012), renal papillary cell carcinoma (*P* = 0.002), liver cancer (*P* < 0.001, P < 0.001), lung adenocarcinoma (*P* = 0.011), pancreatic adenocarcinoma (*P* = 0.002, *P* = 0.018, *P* < 0.001), and gastric adenocarcinoma (*P* = 0.004). Multivariate Cox regression confirmed independent clinical relevance of the signatures in these cancers. ROC curve analyses confirmed superior performance of the signatures to current tumor staging benchmarks. *KDM8* was a potential tumor suppressor down-regulated in liver and pancreatic cancers and an independent prognostic factor. *KDM8* expression was negatively correlated with that of cell cycle regulators. Low *KDM8* expression in tumors was associated with loss of cell adhesion phenotype through *HNF4A* signaling.

**Conclusion:**

Two pan-cancer prognostic signatures of oxygen-sensing genes were identified. These genes can be used for risk stratification in ten diverse cancer types to reveal aggressive tumor subtypes.

**Electronic supplementary material:**

The online version of this article (10.1186/s40880-019-0369-5) contains supplementary material, which is available to authorized users.

## Background

Solid tumors demand a considerable amount of oxygen due to their unique vasculature systems [1]. Rapid neoplastic cell proliferation and overexpression of angiogenic factors leading to the formation of disorganized blood vessels result in insufficient oxygen supply to tumor cells [2, 3]. Hence, there is a requirement for tumors to evolve systems that detect changes in oxygen homeostasis [4]. The discovery of hypoxia-inducible factor (HIF), a key oxygen-sensing gene, represents a quantum leap forward in tumor biology [5, 6]. Its discovery has led to the development of drugs used to treat cancer [7, 8].

In addition to HIF, 2-oxoglutarate (2OG)-dependent oxygenases represent another family of oxygen-sensing proteins. As suggested by the name, this group of enzymes has an absolute requirement for molecular oxygen. They catalyze a range of oxidative modifications, and their activities are affected by nutrient and oxygen availability [9], both of which are altered within the tumor microenvironment. Several members from this gene family have been implicated in cancer. For example, 10–11 translocation 2 is frequently found to be mutated in leukemia [10] and other solid malignancies [11]. In addition, the epigenetic alterations and inactivating mutations of the Jumonji-C domain-containing lysine demethylase (KDM) family are frequently observed in multiple cancers such as multiple myeloma, esophageal squamous cell carcinoma, renal cell carcinoma, breast cancer, colorectal cancer, and glioblastoma [12, 13].

We hypothesized that detecting the expression of 2OG-dependent oxygenases could help predict prognosis in solid malignancies that are characteristically oxygen-deprived. Additionally, we hypothesized this would be applicable to different types of cancer as they share a uniform need to overcome hypoxia for survival. Starting from an initial set of 61 genes encoding 2OG-dependent oxygenases, we developed two prognostic gene signatures, each consisting of a minimal 5 genes that could facilitate risk stratification and predict overall survival (OS) in cancer patients, and further confirmed their prognostic performance through a multi-cohort pan-cancer validation process.

## Methods

### Datasets and processing

Datasets used in this study consist of the expression profiles of 20,752 tumor samples and 881 non-tumor samples that were obtained from The Cancer Genome Atlas (TCGA) [14], International Cancer Genome Consortium (ICGC) [15], and Gene Expression Omnibus (GEO), representing 25 cancer types. The cohort descriptions are listed in Additional file 1. TCGA datasets were downloaded from Broad Institute GDAC Firehose (https://gdac.broadinstitute.org/), which included gene expression profiles of 19,781 tumor samples and 881 non-tumor samples. ICGC datasets were downloaded from the ICGC data portal (https://icgc.org/), which included 729 tumor samples. A GEO dataset was downloaded from the GEO data portal (https://www.ncbi.nlm.nih.gov/geo/), which included 242 tumor samples. TCGA transcriptome datasets were represented as the normalized gene expression RSEM (RNA-seq by expectation maximization) values [16] obtained from GDAC Firehose. ICGC transcriptome datasets were represented as normalized read counts. The GEO dataset was generated by Affymetrix microarray profiling using the Affymetrix Human Genome U133A 2.0 Array [17]. All expression profiles were converted to log_2_(x + 1) scale.

### *KDM8* differential expression analysis

TCGA liver cancer cohort (LIHC; Additional file 1) was used in *KDM8* differential expression analysis. A total of 371 cancer patients in this cohort were median dichotomized into low and high *KDM8* expression groups. To determine differentially expressed genes between the two groups, the Bayes method and linear model were implemented using the R package limma (version 3.8) [18]. *P* values were adjusted using the false discovery rate controlling procedure of Benjamini–Hochberg. Genes with log_2_ fold change of > 1 or < − 1 and adjusted *P* values < 0.05 were considered significant.

### Gene signatures and risk scores

Expression scores for gene signatures 1 and 2 were calculated for each patient by taking the average log_2_ expression values of signature genes. Signature 1 genes: *KDM8, KDM6B, P4HTM, ALKBH4,* and *ALKBH7*. Signature 2 genes: *KDM3A, P4HA1, ASPH, PLOD1,* and *PLOD2*. Tumor hypoxia scores were calculated as the average log_2_ expression values of 52 hypoxia signature genes [19]: *ESRP1, CORO1C, SLC2A1, UTP11, CDKN3, TUBA1B, ENO1, NDRG1, PGAM1, CHCHD2, SLC25A32, SHCBP1, KIF20A, PGK1, BNIP3, ANLN, ACOT7, TUBB6, MAP7D1, YKT6, PSRC1, GPI, PGAM4, GAPDH, MRPL13, SEC61G, VEGFA, MIF, TPI1, MAD2L2, HK2, AK4, CA9, SLC16A1, KIF4A, PSMA7, LDHA, MRPS17, PNP, TUBA1C, HILPDA, LRRC42, TUBA1A, MRGBP, MRPL15, CTSV, ADM, DDIT4, PFKP, P4HA1, MCTS1,* and *ANKRD37*. The risk score for each patient was calculated by taking the sum of Cox regression coefficient for each signature gene multiplied with its corresponding expression value. Nonparametric Spearman’s rank correlation analysis was employed to assess the relationship of expression scores and risk scores with tumor hypoxia (hypoxia score).

### Survival analyses

Cox proportional hazards regression analysis was employed to investigate the association between patient survival and risk factors, e.g., signature 1 or signature 2 score, tumor stage, and other clinical variables. Univariate analyses were performed to determine the influence of individual risk factors on OS. Multivariate analyses were performed by including risk factors that were identified in univariate analyses (*P* < 0.05). Hazard ratios (HR) were determined from Cox models. Cox regression analyses were performed using the R survival (version 2.43-3) [20] and survminer (version 0.4.3) [21] packages. Proportional hazards assumption was supported by a non-significant relationship between scaled Schoenfeld residuals and time using the R survival package. In addition, Kaplan–Meier and log-rank tests were used in univariate analyses of the gene signatures in relation to patient survival and were performed using the survival and survminer packages. Patients were median-dichotomized into low and high-score groups based on median expression scores of signature genes. Differences between high and low-score groups were tested using the log-rank test implemented with the survival package.

Time-dependent receiver operating characteristic (ROC) curve analysis was used to assess the predictive performance of both signatures 1 and 2 in comparison with standard tumor staging parameters. The R survcomp (version 3.8) package [22] was employed to compute time-dependent ROC curves [22].

### Biological enrichment analysis

Analysis of biological pathway enrichment on the 745 differentially expressed genes between *KDM8*-low and -high groups was conducted using GeneCodis against the Kyoto Encyclopedia of Genes and Genomes (KEGG) (https://www.genome.jp/kegg/) and Gene Ontology (GO) databases (http://geneontology.org/) [23]. The Enrichr tool was used to identify transcription factors from the ENCODE database (https://www.encodeproject.org/) as potential regulators of these 745 genes [24, 25].

### *HNF4A* loss-of-function analysis

A total of 148 genes were identified as *HNF4A* targets in the HepG2 hepatoma cell line determined using the Enrichr tool [24, 25]. Differential expression analysis between *HNF4A* wild-type and null mice livers (GSE3126) performed using the GEO2R tool [26] identified 110 differentially expressed genes from the initial 745-gene set identified previously (Fig. 5h). Of these 110 genes, 45 were identified as direct *HNF4A* targets and were down-regulated in the *HNF4A*-null mice (Fig. 5i).

### Somatic mutation identification

Level 3 mutation datasets were downloaded from GDAC (https://gdac.broadinstitute.org/). Kaplan–Meier analysis and log-rank tests were employed to determine the association of somatic mutations, in combination with signature 1 or 2, on OS.

All graphs were generated using the ggplot2 package in R (version 3.1.0) [27].

### Statistical analysis

Comparisons of gene expression levels between tumor and non-tumor samples or between high- and low-score groups were performed using the non-parametric Mann–Whitney–Wilcoxon test implemented in R (version 3.3.3) [28].

## Results

### Multi-cohort analyses revealed two prognostic 2OG-dependent oxygenases signatures

**Fig. 1:**
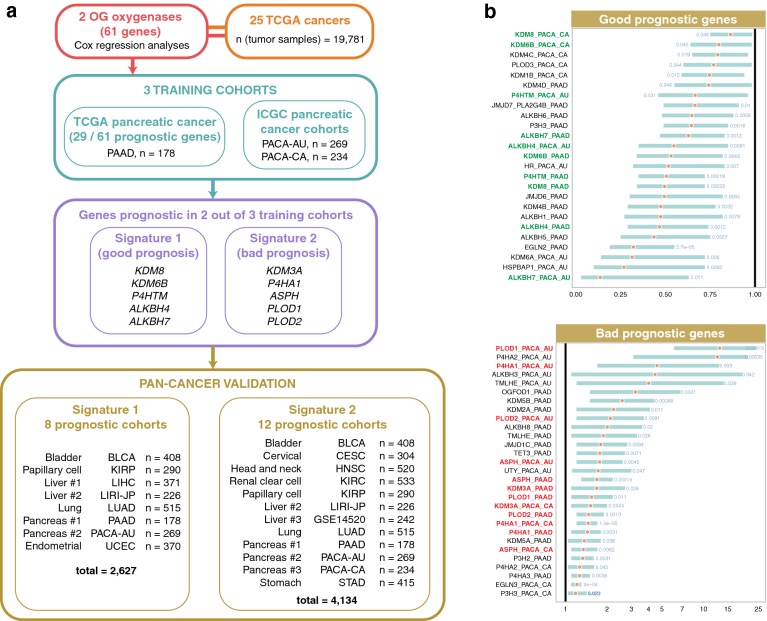
Schematic diagram of the study design and development of signatures derived from 61 2-oxoglutarate-dependent oxygenase genes. **a** Three pancreatic adenocarcinoma cohorts were used to define both signatures 1 and 2. Genes found to be prognostic in univariate Cox regression analysis in 2 out of 3 pancreatic adenocarcinoma cohorts were included in signatures 1 and 2. Signature 1 is a marker of good prognosis and consists of 5 genes (*KDM8, KDM6B, P4HTM, ALKBH4,* and *ALKBH7*). Signature 2 is a marker of adverse prognosis and consists of 5 genes (*KDM3A, P4HA1, ASPH, PLOD1,* and *PLOD2*). Prognosis of both signatures was further confirmed in 10 cancer types using Kaplan–Meier, Cox regression, and receiver operating characteristic analyses. **b** Forest plots of prognostic genes found to be significant by univariate Cox regression analysis in pancreatic adenocarcinoma cohorts abbreviated as PAAD, PACA-AU, and PACA-CA. Genes were separated into two groups, good and bad prognostic genes. Hazard ratios were denoted as red circles, and turquoise bars represent 95% confidence interval. Significant Wald test *P* values are indicated in blue. Y-axes represent gene symbols followed by cohort abbreviations. Signature 1 genes are marked in green. Signature 2 genes are marked in red. Full description of cancers is listed in Additional file 1. 2OG, 2-oxoglutarate; TCGA, The Cancer Genome Atlas; ICGC, International Cancer Genome Consortium

We analyzed the prognostic significance of 61 2OG-dependent oxygenase genes (Additional file 2) in 19,781 tumor samples from multiple TCGA cohorts [14] covering 25 cancer types (Additional file 1). Prognostic genes were defined as those whose expression levels were significantly correlated with patients’ OS. Pancreatic cancer is difficult to treat. Since the highest number of prognostic genes (29 genes) was observed in the pancreatic cancer cohort (PAAD; 178 samples), two additional pancreatic cancer cohorts from ICGC (PACA-AU and PACA-CA; 269 and 234 samples) were used in combination as training cohorts (Fig. 1a). We defined two gene signatures (signatures 1 and 2) as favorable and unfavorable prognostic factors by taking into consideration genes that were significant in univariate Cox regression analyses in 2 out of 3 pancreatic cancer cohorts (Fig. 1a, b). Signature 1 included *KDM8, KDM6B, P4HTM, ALKBH4,* and *ALKBH7*. Likewise, *KDM3A, P4HA1, ASPH, PLOD1,* and *PLOD2* made up signature 2 (Fig. 1a).

**Fig. 2:**
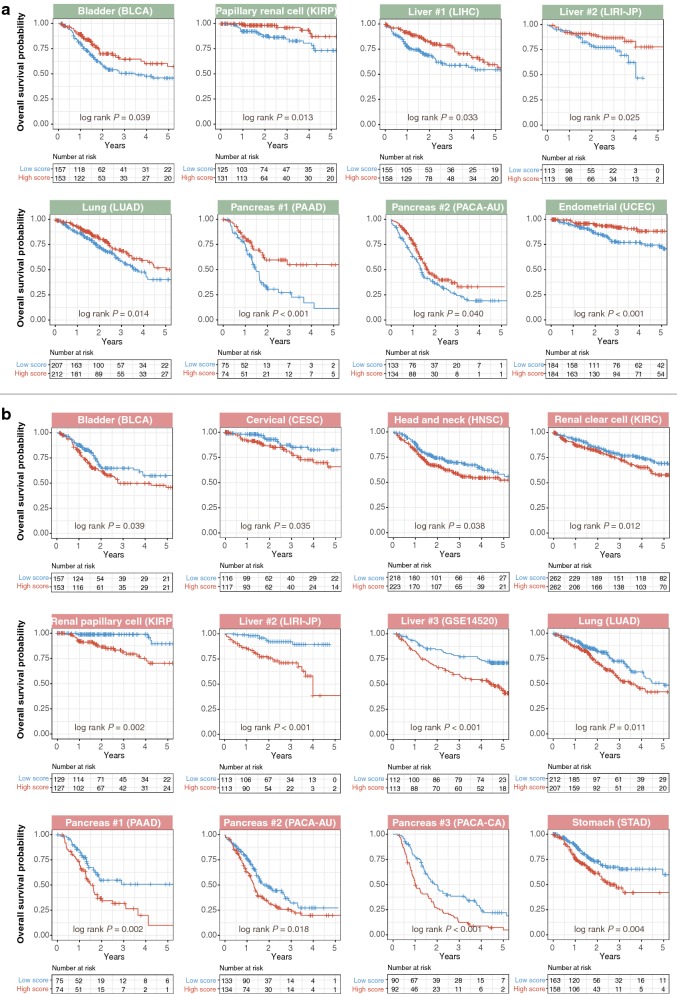
Kaplan–Meier analyses confirming that gene signatures were associated with patients’ overall survival. **a** Validation of signature 1 (green panels) across multiple cancer types. Kaplan–Meier plots of overall survival in cancer patients stratified based on signature 1 mean expression scores. Patients were median-dichotomized into high- and low-score groups. Signature 1 is a marker of good prognosis, and hence patients with high signature 1 scores had high survival rates. **b** Validation of signature 2 (red panels) across multiple cancer types. Kaplan–Meier plots of overall survival in cancer patients stratified based on signature 2 mean expression scores. Patients were median-dichotomized into high- and low-score groups. Signature 2 is a marker of adverse prognosis, and hence patients with high signature 2 scores had low survival rates. *P* values were calculated from the log-rank test. Pancreas #1 = PAAD cohort; Pancreas #2 = PACA-AU cohort; Pancreas #3 = PACA-CA cohort; Liver #1 = LIHC cohort; Liver #2 = LIRI-JP cohort; and Liver #3 = GSE14520 cohort (Additional file 1)

Patients were median-dichotomized based on mean expression scores of signatures 1 and 2 genes. Cox regression analyses revealed that patients with high expression of signature 1 genes had significantly better OS in 6 cancer types (Additional file 3): bladder urothelial carcinoma (BLCA: HR, 0.662; 95% confidence interval [CI] 0.450–0.974; *P* = 0.036), renal papillary cell carcinoma (KIRP: HR, 0.370; 95% CI 0.157–0.871; *P* = 0.023), liver cancer (LIHC: HR, 0.656; 95% CI 0.424–0.915; *P* = 0.048 and LIRI-JP: HR, 0.490; 95% CI 0.259–0.938; *P* = 0.031), lung adenocarcinoma (LUAD: HR, 0.625; 95% CI 0.443–0.879; *P* = 0.007), pancreatic adenocarcinoma (PAAD: HR, 0.454; 95% CI 0.278–0.741; *P* = 0.002), and uterine corpus endometrial carcinoma (UCEC: HR, 0.401; 95% CI 0.229–0.702; *P* = 0.002). Similar results were obtained using log-rank tests, consistent with the fact that signature 1 was a marker of good prognosis (Fig. 2a). In contrast, patients with high expression of signature 2 genes had significantly worse prognosis in 9 cancers: bladder urothelial carcinoma (BLCA: HR, 1.459; 95% CI 1.096–2.137; *P* = 0.042), cervical squamous cell carcinoma and endocervical adenocarcinoma (CESC: HR, 1.972; 95% CI 1.003–3.877; *P* = 0.045), head and neck squamous cell carcinoma (HNSC: HR, 1.479; 95% CI 1.056–2.072; *P* = 0.023), renal clear cell carcinoma (KIRC: HR, 1.483; 95% CI 1.096–2.007; *P* = 0.011), renal papillary cell carcinoma (KIRP: HR, 3.862; 95% CI 1.565–9.526; *P *= 0.003), liver cancer (LIRI-JP: HR, 5.271; 95% CI 2.429–11.440; *P* < 0.001 and GSE14520: HR, 2.285; 95% CI 1.458–3.580; *P* < 0.001), lung adenocarcinoma (LUAD: HR, 1.562; 95% CI 1.116–2.188; *P* = 0.009), pancreatic adenocarcinoma (PAAD: HR, 1.969; 95% CI 1.217–3.186; *P* = 0.006), and gastric adenocarcinoma (STAD: HR, 1.725; 95% CI 1.142–2.605; *P* = 0.009) (Fig. 2b and Additional file 3).

### Cross-platform subgroup and multivariate analyses confirmed the validity of signatures 1 and 2 as independent prognostic factors

**Fig. 3:**
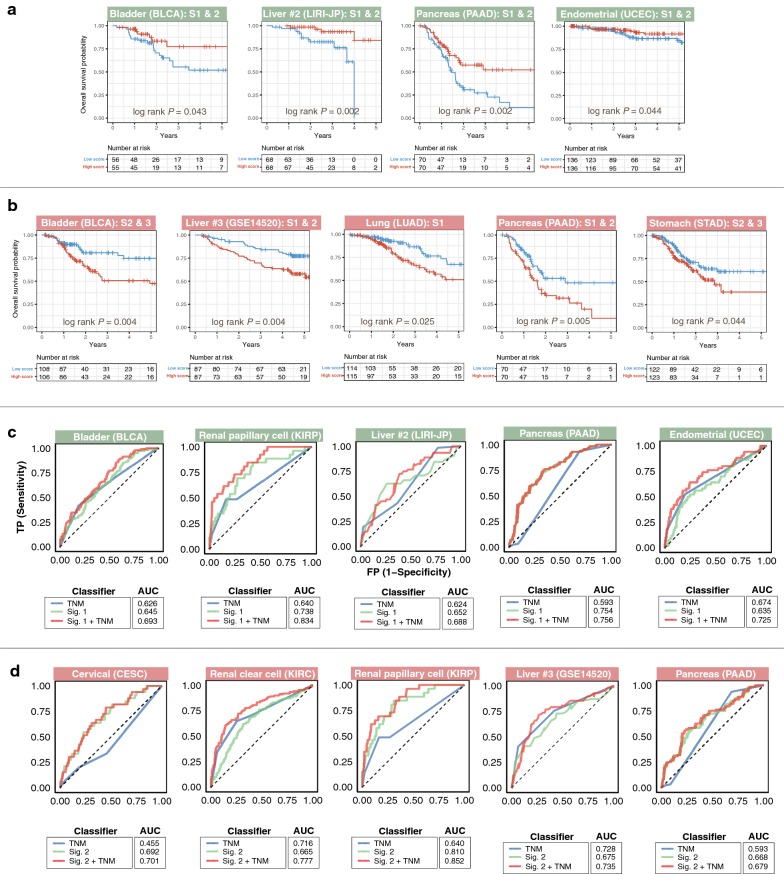
Tumor subgroup analyses and evaluation of prognosis predictive performance of gene signatures across different malignant grades. Kaplan–Meier plots show independence of **a** signature 1 (green panels) and **b** signature 2 (red panels) over the current TNM staging system in predicting prognosis in different cancer cohorts. Patients were sub-grouped according to TNM stages and further stratified using either signature 1 or signature 2 scores. Both signatures successfully identified high-risk patients in different TNM stages. *P* values were calculated from the log-rank test. Analysis of specificity and sensitivity of **c** signature 1 (green panels) and **d** signature 2 (red panels) in predicting prognosis in different cancer cohorts using receiver operating characteristic (ROC) curves. Plots depict comparison of ROC curves of signature 1 or 2 and clinical TNM staging. Both signatures demonstrate incremental values over the current TNM staging system. AUC: area under the curve. TNM: tumor, node, metastasis staging. Liver #2 = LIRI-JP cohort and Liver #3 = GSE14520 cohort (Additional file 1). Representative plots are depicted in this figure. Additional plots are available in Additional file 4

To assess the independence of signatures 1 and 2 over current tumor staging systems, we performed subgroup analyses of their prognostic effects in patients with early (stages I and/or II), intermediate (stages II and/or III), and late (stages III and/or VI) cancer stages. Kaplan–Meier analyses revealed that signature 1 successfully identified high-risk (low-expression score) and low-risk (high-expression score) patients with early (bladder urothelial carcinoma, liver cancer, lung adenocarcinoma, pancreatic adenocarcinoma, and uterine corpus endometrial carcinoma), intermediate (liver cancer and uterine corpus endometrial carcinoma), and late (renal papillary cell carcinoma) disease stages (Fig. 3a and Additional file 4A). Signature 2 was also independent of disease stage as it successfully predicted survival in early (liver cancer, lung adenocarcinoma, and pancreatic adenocarcinoma), intermediate (bladder urothelial carcinoma, liver cancer, and gastric adenocarcinoma), and late (bladder urothelial carcinoma, head and neck squamous cell carcinoma, renal papillary cell carcinoma, liver cancer, and gastric adenocarcinoma) stages (Fig. 3b and Additional file 4B).

ROC analyses were employed to determine the predictive performance (sensitivity and specificity) of signatures 1 and 2 on 5-year OS. Signature 1 performed the best, as measured by area under the curve (AUC), in pancreatic adenocarcinoma (PAAD: AUC = 0.754) followed by renal papillary cell carcinoma (KIRP: AUC = 0.738), liver cancer (LIRI-JP: AUC = 0.652 and LIHC: AUC = 0.613), bladder urothelial carcinoma (BLCA: AUC = 0.645), uterine corpus endometrial carcinoma (UCEC: AUC = 0.635), and lung adenocarcinoma (LUAD: AUC = 0.625) (Fig. 3c and Additional file 4C). Signature 2 performance in renal papillary cell carcinoma (KIRP: AUC = 0.810) was the best, followed by cervical squamous cell carcinoma and endocervical adenocarcinoma (CESC: AUC = 0.692), liver cancer (GSE14520: AUC = 0.675 and LIRI-JP: AUC = 0.625), pancreatic adenocarcinoma (PAAD: AUC = 0.668), renal clear cell carcinoma (KIRC: AUC = 0.665), head and neck squamous cell carcinoma (HNSC: AUC = 0.632), lung adenocarcinoma (LUAD: AUC = 0.623), gastric adenocarcinoma (STAD: AUC = 0.618), and bladder urothelial carcinoma (BLCA: AUC = 0.605) (Fig. 3d and Additional file 4D). Performance of both signatures 1 and 2 was superior to current tumor-node-metastasis (TNM) staging except for the following: signature 1 in liver cancer, lung adenocarcinoma, and uterine corpus endometrial carcinoma and signature 2 in bladder urothelial carcinoma, renal clear cell carcinoma, liver cancer, and lung adenocarcinoma (Fig. 3c, d and Additional file 4C, D). Remarkably, when used in combination with TNM staging, both signatures consistently outperformed each of the individual classifiers, reinforcing their incremental prognostic values (Fig. 3c, d and Additional file 4C, D). Significantly, while TNM staging could not predict outcome in cervical squamous cell carcinoma and endocervical adenocarcinoma patients (CESC: AUC = 0.455), signature 2 sufficiently served as an adverse prognostic factor (CESC: AUC = 0.692) (Fig. 3d).

Univariate Cox regression analyses revealed that TNM stage was associated with patient survival in different cancer types except for cervical squamous cell carcinoma and endocervical adenocarcinoma, and pancreatic adenocarcinoma (Additional file 3). This was expected given the low AUC values for TNM stage in both pancreatic adenocarcinoma (PAAD: AUC = 0.593) and cervical squamous cell carcinoma and endocervical adenocarcinoma (CESC: AUC = 0.455), suggesting that current TNM staging system for these cancers are inadequate (Fig. 3c, d). Multivariate Cox regression analyses after adjusting for TNM stage showed that signatures 1 and 2 remained significantly associated with survival (Additional file 3). For 2 liver cancer cohorts, we considered additional clinicopathological features. The GSE14520 cohort consisted of Chinese patients with hepatitis B-associated hepatocellular carcinoma [17], whereas LIRI-JP was a Japanese-based cohort of mixed etiology [29]. Tumor size, cirrhosis, TNM stage, Barcelona Clinic Liver Cancer (BCLC) stage, and alpha-fetoprotein (AFP) levels were all significantly associated with survival in the GSE14520 cohort; tumor size could also predict survival in the LIRI-JP cohort (Additional file 3). When these significant covariates along with signatures 1 or 2 were included in multivariate Cox models, the signatures remained significant risk factors: signature 1 (LIRI-JP: HR, 0.541; 95% CI 0.283–0.904; *P* = 0.043) and signature 2 (LIRI-JP: HR, 4.539, 95% CI 2.055–10.029; *P *< 0.001 and GSE14520: HR, 2.012; 95% CI 1.267–3.195; *P* = 0.003) (Additional file 3). These results highlight the potentially superior prognostic ability of our signatures: signatures 1 and 2 identified high- and low-risk patients in 8 and 12 independent cohorts covering 10 cancer types (Fig. 1a).

### Significance of somatic mutations in risk-stratified patients

Patients were risk stratified into low- and high-risk groups using signatures 1 and 2. For signature 1, high-risk patients had significantly lower expression levels of good prognosis genes *ALKBH4, ALKBH7, KDM8, KDM6B,* and *P4HTM* (Additional file 5A). In contrast, high-risk patients as stratified by signature 2 had significantly higher expression levels of adverse prognosis genes *ASPH, KDM3A, P4HA1, PLOD1,* and *PLOD2* (Additional file 5B). To ascertain the relationship between tumor hypoxia and expression of signature genes, hypoxia scores were computed for each patient as mean expression values (log_2_) of 52 hypoxia signature genes [19]. Signature 1 expression scores in patients negatively correlated with hypoxia score (Additional file 6A). Since tumor hypoxia is associated with distant metastasis, recurrence, and reduced therapeutic response [30], high expression of signature 1 genes (low hypoxia score) was correlated with less advanced disease states consistent with it being a marker of good prognosis (Additional file 6A).

Conversely, signature 2 scores positively correlated with tumor hypoxia and hence poor survival outcomes (Additional file 7A). We anticipated that patients’ individual risks of death, as determined from signatures 1 and 2, would positively correlate with tumor hypoxia. Indeed, the risk score for each patient, as calculated by taking the sum of Cox regression coefficient for each of the individual genes multiplied with its corresponding expression value [31], was correlated with the hypoxia score (Additional files 6B, 7B). Hence, high-risk patients had more hypoxic tumors, suggesting that our gene signatures are efficient and adequate in predicting death.

To ascertain the association between patients’ risks, as determined by our gene signatures, and somatic mutations, we retrieved the five most commonly mutated genes for each cancer. Mutations in *PCDHA1*, a cell adhesion gene from the cadherin superfamily, were associated with short survival in bladder urothelial carcinoma (BLCA: HR, 1.649; 95% CI 1.058–2.569; *P *= 0.027) and gastric adenocarcinoma (STAD: HR, 1.525; 95% CI 1.007–2.307; *P *= 0.046) but with prolonged survival in uterine corpus endometrial carcinoma (UCEC: HR, 0.516; 95% CI 0.272–0.978; *P *= 0.042) (Additional file 3). Mutations in another gene from the protocadherin alpha cluster, *PCDHA2*, were also associated with adverse outcomes in gastric adenocarcinoma (STAD: HR, 1.604; 95% CI 1.061–2.427; *P *= 0.025) (Additional file 3). Mutations in *TTN* and the tumor suppressor *TP53* were associated with short survival in bladder urothelial carcinoma (BLCA: HR, 1.610; 95% CI 1.091–2.376; *P *= 0.016) and uterine corpus endometrial carcinoma (UCEC: HR, 1.780; 95% CI 1.025–3.090; *P *= 0.041) (Additional file 3). Interestingly, another tumor suppressor *PTEN*, when mutated, was linked to better outcomes in uterine corpus endometrial carcinoma (UCEC: HR, 0.427; 95% CI 0.234–0.781; *P *= 0.006) (Additional file 3). Similar observations were made for a lipid kinase gene *PIK3CA* in uterine corpus endometrial carcinoma (UCEC: HR, 0.362; 95% CI 0.190–0.689; *P *= 0.002) (Additional file 3). Likewise, *MUC4* mutations prolonged survival in renal clear cell carcinoma patients (KIRC: HR, 0.570; 95% CI 0.370–0.880; *P *= 0.012) (Additional file 3), an observation that is consistent with another study [32].

**Fig. 4:**
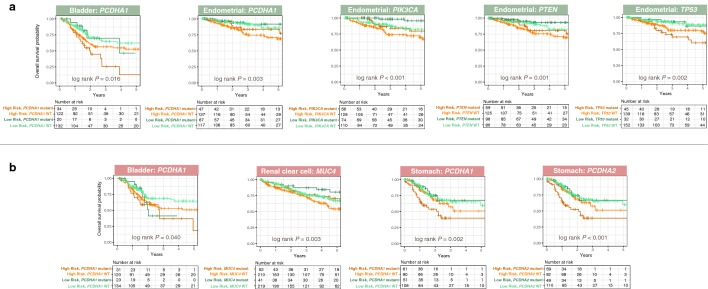
Relationship between patients’ risks as determined by gene signatures and common genetic mutations. Patients were median-stratified into low or high-risk groups using **a** signature 1 (green panels) and **b** signature 2 (red panels). Since signature 1 is a marker of good prognosis, high-risk patients had a lower mean expression of signature 1 genes. Signature 2 is a marker of poor prognosis, hence high-risk patients had a higher mean expression of signature 2 genes. Kaplan–Meier plots depict combined relation of somatic mutations with signatures 1 or 2 on overall survival in cancer patients. *P* values were calculated from the log-rank test

Multivariate Cox regression analyses on signatures 1 and 2 while controlling for significant somatic mutation variables revealed that the gene signatures were independent survival predictors for bladder urothelial carcinoma (signature 1: HR, 0.686; 95% CI 0.466–0.912; *P *= 0.047 and signature 2: HR, 1.411; 95% CI 1.062–2.070; *P *= 0.048), renal clear cell carcinoma (signature 2: HR, 1.520; 95% CI 1.123–2.056; *P *= 0.007), gastric adenocarcinoma (signature 2: HR, 1.800; 95% CI 1.184–2.737; *P *= 0.006), and uterine corpus endometrial carcinoma (signature 1: HR, 0.519; 95% CI 0.293–0.920; *P *= 0.024) (Additional file 3). Signatures 1 or 2 and mutation status were collectively associated with OS (Fig. 4). In bladder urothelial carcinoma, high-risk patients (low signature 1 score) harboring mutant alleles of *PCDHA1* had ~ 50% increased mortality at 5 years compared to low-risk patients (high signature 1 score) with wild-type *PCDHA1* (*P* = 0.016; Fig. 4). Although results were less dramatic for *PCDHA1* and signature 2, we still observed a ~ 25% elevated mortality at 5 years for these two patient groups with bladder urothelial carcinoma (*P* = 0.040; Fig. 4). In gastric adenocarcinoma, high-risk patients (high signature 2 scores) with mutant *PCDHA1* had the worst outcomes (*P* = 0.002; Fig. 4). Conversely, *PCDHA1* mutation was associated with good prognosis in uterine corpus endometrial carcinoma, hence high-risk patients with wild-type *PCDHA1* had the lowest survival rates while survival was prolonged by ~ 20% in low-risk patients with mutant *PCDHA1* (*P* = 0.003; Fig. 4). *PIK3CA* (*P* < 0.001*)* and *PTEN* mutations (*P* = 0.001) were associated with good outcomes in uterine corpus endometrial carcinoma (Fig. 4). Mutations in another cadherin gene *PCDHA2* when considered alongside signature 2 were also associated with survival in gastric adenocarcinoma (*P* < 0.001; Fig. 4). Survival rates were reduced by ~ 37% in high-risk patients with mutant *PCDHA2* (Fig. 4). Joint relation between *TP53* mutations and signature 1 significantly influenced survival in uterine corpus endometrial carcinoma (*P* = 0.002; Fig. 4). Since *MUC4* mutations were associated with good outcomes, survival rates were the lowest in high-risk patients (high signature 2 scores) with wild-type *MUC4* (*P* = 0.003; Fig. 4).

### Tumor suppressive roles of *KDM8* through cell cycle regulation and cell adhesion maintenance

**Fig. 5:**
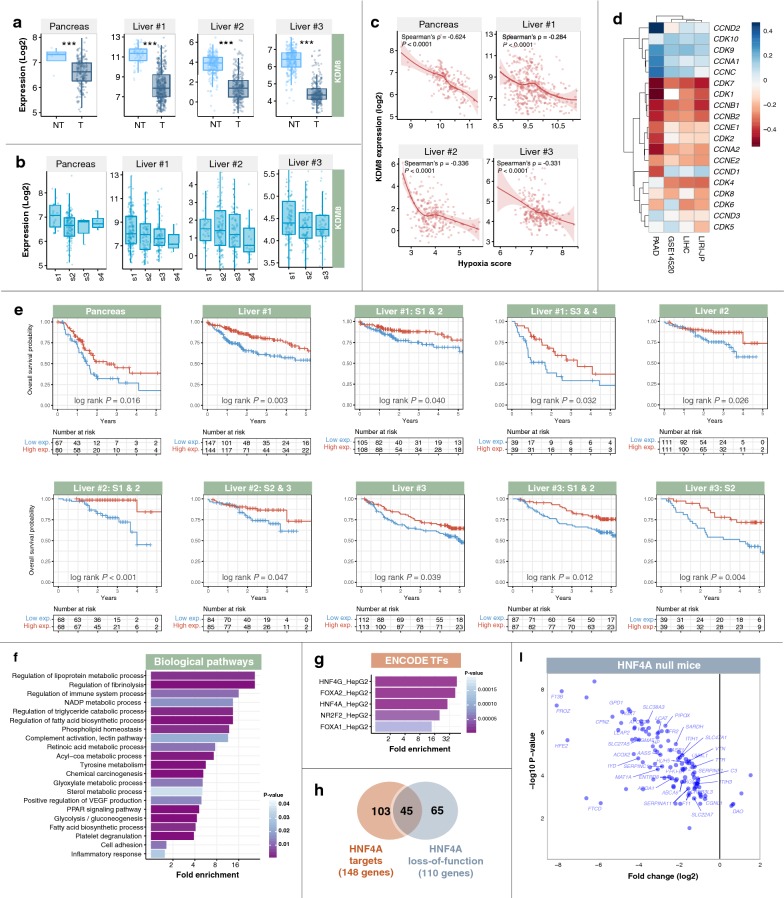
Putative tumor suppressive functions of *KDM8* occur through processes related to cell cycle regulation and cell adhesion. **a** Expression of *KDM8* was significantly lower in tumor (T) samples than in non-tumor (NT) samples in liver and pancreatic cancer cohorts. Mann–Whitney–Wilcoxon tests were used to compare T and NT samples. Asterisks represent significant *P* values: *** < 0.0001. **b** Expression levels of *KDM8* decreased with disease progression and malignant grade in liver and pancreatic cancer cohorts. **c** Significant negative correlation between patients’ *KDM8* expression and tumor hypoxia (hypoxia score) in liver and pancreatic cancer cohorts. **d** Correlation between *KDM8* expression and canonical cell cycle regulators in patients with liver or pancreatic cancers. A majority of genes involved in cell-cycle regulation are negatively correlated with *KDM8* expression. Liver #1 = LIHC cohort; Liver #2 = LIRI-JP cohort; and Liver #3 = GSE14520 cohort (Additional file 1). **e** Kaplan–Meier analysis of patients stratified by *KDM8* expression. Patients were median-dichotomized into low- and high-expression groups. Patients with low *KDM8* expression had significantly shorter overall survival. This was consistent in patients analyzed as a full cohort or sub-categorized according to TNM stage. Liver #1 = LIHC cohort; Liver #2 = LIRI-JP cohort; and Liver #3 = GSE14520 cohort (Additional file 1). **f** Patients were median-stratified according to KDM8 expression. Differential expression analysis between *KDM8*-high- and -low groups in liver cancer cohorts revealed 745 differentially expressed genes (DEGs; fold-change > 2 or < − 2). Enrichment of biological pathways associated with DEGs, which include processes related to cell adhesion, inflammation, metabolism, and signal transduction pathways in cancer. **g** Enrichment of transcription factors (TFs) from the ENCODE database that are potential regulators of *KDM8* DEGs. These TFs were predicted to bind near *KDM8* DEGs. **h** Venn diagram depicts the overlap between *HNF4A* targets (as identified by ENCODE chromatin-immunoprecipitation sequencing dataset) and genes affected by *HNF4A* loss-of-function (as identified in HNF4A-null mice). Of the 745 DEGs, 148 were identified as direct *HNF4A* targets, and 110 genes were affected by *HNF4A* loss-of-function. In the Venn intersection, 45 genes were both *HNF4A* targets and altered in *HNF4A*-null mice. **i** Scatter plot depicts expression patterns of 110 genes affected by *HNF4A* loss-of-function. Gene names of the 45 *HNF4A* targets are annotated on the plot. A majority of *KDM8*-associated genes were down-regulated in the *HNF4A*-null mice

Of all the signature genes, *KDM8* was identified as one of the most down-regulated genes in tumors (Fig. 5a). Patients with high *KDM8* levels had a significantly lower risk of death in pancreatic and liver cancer cohorts (Fig. 5e). Prognostic significance of *KDM8* was also independent of tumor stage (Fig. 5e). *KDM8* expression decreased as tumor malignant grade increased in that stage 1 tumors had the highest median *KDM8* values (Fig. 5b). Moreover, *KDM8* expression was negatively correlated with hypoxia score, indicating that patients with low levels of *KDM8* had more hypoxic tumors and poorer survival outcomes (Fig. 5c). Together, these observations suggest that *KDM8* may function as a tumor suppressor. This hypothesis is corroborated by an independent report on the role of *KDM8* in cell cycle regulation [33]. Indeed, we observed that *KDM8* expression was negatively correlated with the expression levels of canonical cell cycle genes: cyclins (*CCNA2, CCNB1, CCNB2, CCND1, CCNE1,* and *CCNE2*) and cyclin-dependent kinases (*CDK1, CDK2, CDK4, CDK6, CDK7,* and *CDK8*), which were consistent across all liver and pancreatic cancer cohorts (Fig. 5d). This implied that *KDM8* is required for tight control of the cell cycle machinery and its reduction may lead to aberrant proliferation commonly seen in cancer cells.

To ascertain the biological consequences of deregulated *KDM8* expression, we conducted differential expression analysis on liver cancer patients categorized into *KDM8*-low and -high groups. A total of 745 genes were differentially expressed (DEGs) between the two groups (fold change > 2 or < − 2, *P* < 0.05) (Additional file 8). Significant enrichments of biological pathways involved in metabolism, immune regulation, *VEGF* production, inflammation, and cell adhesion were observed (Fig. 5f and Additional file 9). Furthermore, DEGs were overexpressed as targets of *HNF4A, HNF4G, FOXA1, FOXA2,* and *NR2F2* transcription factors (TFs) (Fig. 5g). These TFs play central roles in cell polarity maintenance and epithelial differentiation [26, 34, 35], hence down-regulation of *KDM8* may drive epithelial–mesenchymal transition (EMT) and tumor progression. *HNF4A* is a key TF responsible for regulating a myriad of hepatic functions including cell junction assembly [26, 36]. Pathway analysis of *KDM8* DEGs revealed enrichment of processes related to cell adhesion, suggesting potential crosstalk between *KDM8* and *HNF4A*. Of the 745 DEGs, analysis on a hepatoma-based *HNF4A* chromatin immunoprecipitation-sequencing dataset demonstrated that 148 genes were directly bound by *HNF4A* [37]. To further reinforce the interplay between *HNF4A* and *KDM8*, we observed that 110 of the 745 DEGs were overrepresented in *HNF4A*-null mice [26] and 45 of these genes were direct *HNF4A* targets (Fig. 5h). Differential expression analysis between *HNF4A*-deficient and wild-type mice showed that a majority of the 45 genes were down-regulated, as expected, suggesting that *HNF4A* directly activates their gene expression, many of which are involved in a multitude of cell adhesion processes (Fig. 5i).

## Discussion

The present multi-cohort retrospective study identified two novel pan-cancer prognostic gene signatures derived from oxygen-sensing genes. Cross-platform validations confirmed prognosis in 10 cancer types to collectively include 6761 patients spanning 20 diverse cohorts (Fig. 1a). The gene signatures had opposing prognostic values: signature 1 is a marker of good prognosis, whereas signature 2 is associated with poor outcomes. The key strengths of our signatures as powerful prognostic tools are (1) pan-cancer utility, (2) involvement of a mere 5 genes each that provide continuous assessment of death risks, and (3) superiority over current TNM staging. Our results suggest that dysregulated oxygen sensing in diverse cancer types may activate other oncogenic pathways such as the loss of cell polarity and cell cycle regulation, which collectively influenced clinical outcomes in patients.

Anti-tumorigenic functions have been reported for several genes from signature 1. Loss of *KDM6B* resulted in more aggressive pancreatic ductal adenocarcinoma [38]. In colorectal cancer, high *KDM6B* expression predicted good prognosis, and knock-down of *KDM6B* was associated with augmented cell proliferation and inhibited apoptosis [39]. Yet, *KDM6B* function is enigmatic. Others have reported that high *KDM6B* expression is associated with increased metastasis and invasion of renal clear cell carcinoma [40]. *KDM6B* also promotes TGF-β-induced EMT and invasiveness in breast cancer [41]. While we could neither confirm nor deny the validity of these studies, it is striking that our observation of favorable prognosis associated with high *KDM6B* expression in pancreatic ductal adenocarcinoma was consistent with the report from Yamamoto et al. [38]. We also did not observe any prognostic significance of signature 1, which includes *KDM6B*, in either breast or renal clear cell cancer, which indirectly substantiates findings from two other reports on *KDM6B* not being a marker of good prognosis [40, 41]. Several other gene signatures have been reported for gastrointestinal cancers [42–46]. Interestingly, there is no overlap between our signature genes and those identified in these studies. This is perhaps not surprising since our signatures were identified based on prognostic information in pancreatic cancer, whereas those studies employed very different approaches for gene signature discovery.

*KDM8* is a gene associated with favorable prognosis. Our results suggest determinative crosstalk between *KDM8* and *HNF4A*, particularly in the context of morphogenesis, cell adhesion, maintenance of cell polarity, and epithelial formation. Moreover, 5-year survival rates dropped to ~ 12% in bladder cancer patients with low expression of signature 1 genes (high-risk), which included *KDM8* and *PCDHA1* mutations. Additive effects conferred by mutations in this cell-adhesion protein supports the hypothesis that *KDM8* is likely a tumor suppressor and down-regulation of this gene may lead to a loss of epithelial phenotype and cell adhesion to promote cancer invasion. Additionally, loss of *KDM8* expression is correlated with increased expression of cell cycle genes that may contribute to deranged cell cycle regulation and tumor progression (Fig. 5d). Like *KDM6B*, the function of *KDM8* appears to be cell type-dependent. While *KDM8* expression is down-regulated in liver and pancreatic tumor samples compared to adjacent non-tumor samples (Fig. 5a), it is overexpressed in breast cancer to induce EMT and invasion [47]. Nonetheless, *KDM8* roles are not limited to cell cycle regulation. *KDM8* exerts tumor suppressive functions in hematopoietic cancer by mediating DNA repair [48]. Collectively, imbalance in the Jumonji-C subfamily of lysine demethylases such as *KDM8* and *KDM6B* is likely to result in broad-ranging but cell type-specific biological effects.

## Conclusions

Overall, our gene signatures would enhance decision making in clinic by stratifying patients according to their tumor biology. This may maximize treatment efficacy and prolong lifespan by directing resources to those most in need. This technology may be incorporated into existing diagnostic pathways to achieve a more individualized standard of care by revealing molecular changes that allow further discrimination of otherwise similarly staged tumors. As each signature only consists of 5 genes, we anticipate that they can be implemented immediately, even in modestly sized centers that are using PCR-based technology. Consequently, therapeutic options can be allocated more decisively based upon this additional personalized information to ensure that patients with the most aggressive cancers get the most robust treatments.

## Additional files

**Additional file 1.** Cancer cohort descriptions.

**Additional file 2.** List of 61 2OG-dependent oxygenases.

**Additional file 3.** Univariate and multivariate Cox proportional hazards analysis of risk factors associated with overall survival in multiple cancers. Univariate values of TNM stage were in accordance with our previous report utilizing TCGA datasets [4].

**Additional file 4.** Additional tumor subgroup analyses and evaluation of prognosis predictive performance of gene signatures across different malignant grades. Kaplan–Meier plots show independence of (A) signature 1 (green panels) and (B) signature 2 (red panels) over current TNM staging system in predicting prognosis in different cancer cohorts. Patients were sub-grouped according to TNM stages and further stratified using either signature 1 or signature 2 scores. Both signatures successfully identified high-risk patients in different TNM stages. *P* values were calculated from the log-rank test. Analysis of specificity and sensitivity of (C) signature 1 (green panels) and (D) signature 2 (red panels) in predicting prognosis in different cancer cohorts using receiver operating characteristic (ROC) curves. Plots depict comparison of ROC curves of signature 1 or 2 and clinical TNM staging. Both signatures demonstrated incremental values over current TNM staging system. AUC: area under the curve. TNM: tumor, node, metastasis staging. Liver #1 = LIHC cohort; Liver #2 = LIRI-JP cohort and Liver #3 = GSE14520 cohort (Additional file 1).

**Additional file 5.** Distribution of expression of signature genes in low- and high-risk patients. (A) signature 1 (green panels) and (B) signature 2 (red panels). Patients were median-stratified into low- and high-risk groups based on mean expression scores of signature genes. Box plots depict expression distribution of each of the 5 genes in both signatures in these two patient groups. (A) Since signature 1 is a marker of good prognosis, high-risk patients show significantly lower expression of individual signature genes. (B) In contrast, signature 2 is a marker of poor prognosis, hence high-risk patients show significantly higher expression of individual signature genes. Nonparametric Mann–Whitney–Wilcoxon tests were used to compare low- and high-risk patients. Asterisks represent significant *P* values: * < 0.01, ** < 0.001 and *** < 0.0001. LR = low risk. HR = high risk.

**Additional file 6.** Correlation of patients’ risk scores derived from signature 1 with tumor hypoxia. (A) Significant negative correlation between signature 1 expression scores and tumor hypoxia. (B) Significant positive correlation between signature 1 risk scores and tumor hypoxia. Calculations of expression scores, risk scores, and hypoxia scores are explained in the methods. Liver #1 = LIHC cohort and Liver #2 = LIRI-JP cohort.

**Additional file 7.** Correlation of patients’ risk scores derived from signature 2 with tumor hypoxia. (A) Significant positive correlation between signature 2 expression scores and tumor hypoxia. (B) Significant positive correlation between signature 2 risk scores and tumor hypoxia. Calculations of expression scores, risk scores and hypoxia scores are explained in the methods. Liver #2 = LIRI-JP cohort and Liver #3 = GSE14520 cohort.

**Additional file 8.** Differentially expressed genes between *KDM8*-high and -low groups in the liver cancer cohort (LIHC).

**Additional file 9.** Significantly enriched biological pathways of differentially expressed genes.

## Abbreviations

HIF: hypoxia inducible factor
2OG: 2-oxoglutarate
OS: overall survival
KDM: Jumonji-C domain-containing lysine demethylase
TCGA: The Cancer Genome Atlas
ICGC: International Cancer Genome Consortium
GEO: Gene Expression Omnibus
RSEM: RNA-seq by expectation maximization
HR: hazard ratio
CI: confidence interval
ROC: receiver operating characteristic
AUC: area under the curve
KEGG: Kyoto Encyclopedia of Genes and Genomes
GO: gene ontology
BCLC: Barcelona Clinic Liver Cancer
TNM: tumor, node, metastasis
EMT: epithelial–mesenchymal transition
DEG: differentially expressed gene
TF: transcription factor
AFP: alpha-fetoprotein
