# Supplementary material for: Dual prognostic role of 2-oxoglutarate-dependent oxygenases in ten cancer types: implications for cell cycle regulation and cell adhesion maintenance
Source: Cancer Commun (Lond). 2019 Apr 29;39:23. doi: 10.1186/s40880-019-0369-5 (PMC6489267; doi:10.1186/s40880-019-0369-5)
Supplement: Supplementary file 1 — Additional file 1. Cancer cohort descriptions. [file 40880_2019_369_MOESM1_ESM.docx]

| **Additional File 1. Cancer cohort descriptions.** | | | | |
| --- | --- | --- | --- | --- |
|  | | | | |
| **Cohort** | **No. of non-tumor samples** | **No. of tumor samples** | **Cancer type** | **Source** |
| BLCA | 19 | 408 | Bladder urothelial carcinoma | TCGA |
| BRCA | 112 | 10,939 | Breast invasive carcinoma | TCGA |
| CESC | 3 | 304 | Cervical squamous cell carcinoma and endocervical adenocarcinoma | TCGA |
| CHOL | 9 | 36 | Cholangiocarcinoma | TCGA |
| COAD | 41 | 285 | Colon adenocarcinoma | TCGA |
| ESCA | 11 | 184 | Esophageal carcinoma | TCGA |
| GBM | 5 | 153 | Glioblastoma multiforme | TCGA |
| GBMLGG | 5 | 669 | Glioma | TCGA |
| HNSC | 44 | 520 | Head and neck squamous cell carcinoma | TCGA |
| KICH | 25 | 66 | Kidney chromophobe | TCGA |
| KIPAN | 129 | 889 | Pan-kidney cancer | TCGA |
| KIRC | 72 | 533 | Renal clear cell carcinoma | TCGA |
| KIRP | 32 | 290 | Renal papillary cell carcinoma | TCGA |
| LIHC (Liver cohort 1) | 50 | 371 | Hepatocellular carcinoma | TCGA |
| LUAD | 59 | 515 | Lung adenocarcinoma | TCGA |
| LUSC | 51 | 501 | Lung squamous cell carcinoma | TCGA |
| PAAD (Pancreas cohort 1) | 4 | 178 | Pancreatic adenocarcinoma | TCGA |
| PCPG | 3 | 179 | Pheochromocytoma and paraganglioma | TCGA |
| PRAD | 52 | 497 | Prostate adenocarcinoma | TCGA |
| SARC | 2 | 259 | Sarcoma | TCGA |
| STAD | 35 | 415 | Stomach adenocarcinoma | TCGA |
| STES | 46 | 599 | Stomach and esophageal carcinoma | TCGA |
| THCA | 59 | 501 | Thyroid carcinoma | TCGA |
| THYM | 2 | 120 | Thymoma | TCGA |
| UCEC | 11 | 370 | Uterine corpus endometrial carcinoma | TCGA |
| LIRI-JP (Liver cohort 2) | NA | 226 | Hepatocellular carcinoma | ICGC |
| GSE14520 (Liver cohort 3) | NA | 242 | Hepatocellular carcinoma | GEO |
| PACA-AU (Pancreas cohort 2) | NA | 269 | Pancreatic adenocarcinoma | ICGC |
| PACA-CA (Pancreas cohort 3) | NA | 234 | Pancreatic adenocarcinoma | ICGC |

Abbreviations: TCGA, the Cancer Genome Atlas; ICGC, International Cancer Genome Consortium; GEO, Gene Expression Omnibus; NA, not applicable.
