# Supplementary material for: Dual prognostic role of 2-oxoglutarate-dependent oxygenases in ten cancer types: implications for cell cycle regulation and cell adhesion maintenance
Source: Cancer Commun (Lond). 2019 Apr 29;39:23. doi: 10.1186/s40880-019-0369-5 (PMC6489267; doi:10.1186/s40880-019-0369-5)
Supplement: Supplementary file 2 — Additional file 2. List of 61 2OG-dependent oxygenases. [file 40880_2019_369_MOESM2_ESM.docx]

| **Additional File 2. List of 61 genes encoding 2-oxoglutarate oxygenases** | |
| --- | --- |
|  | |
| **Gene Symbol** | **Description** |
| ALKBH1 | alkB homolog 1, histone H2A dioxygenase |
| ALKBH2 | alkB homolog 2, alpha-ketoglutarate dependent dioxygenase |
| ALKBH3 | alkB homolog 3, alpha-ketoglutaratedependent dioxygenase |
| ALKBH4 | alkB homolog 4, lysine demethylase |
| ALKBH5 | alkB homolog 5, RNA demethylase |
| ALKBH6 | alkB homolog 6 |
| ALKBH7 | alkB homolog 7 |
| ALKBH8 | alkB homolog 8, tRNA methyltransferase |
| ASPH | aspartate beta-hydroxylase |
| BBOX1 | gamma-butyrobetaine hydroxylase 1 |
| EGLN1 | egl-9 family hypoxia inducible factor 1 |
| EGLN2 | egl-9 family hypoxia inducible factor 2 |
| EGLN3 | egl-9 family hypoxia inducible factor 3 |
| FTO | fat mass and obesity associated alpha-ketoglutarate dependent dioxygenase |
| HR | lysine demethylase and nuclear receptor corepressor |
| HSPBAP1 | HSPB1 associated protein 1 |
| JARID2 | jumonji and AT-rich interaction domain containing 2 |
| JMJD1C | jumonji domain containing 1C |
| JMJD4 | jumonji domain containing 4 |
| JMJD6 | arginine demethylase and lysine hydroxylase |
| JMJD7 | jumonji domain containing 7 |
| JMJD8 | jumonji domain containing 8 |
| KDM1A | lysine demethylase 1A |
| KDM1B | lysine demethylase 1B |
| KDM2A | lysine demethylase 2A |
| KDM2B | lysine demethylase 2B |
| KDM3A | lysine demethylase 3A |
| KDM3B | lysine demethylase 3B |
| KDM4A | lysine demethylase 4A |
| KDM4B | lysine demethylase 4B |
| KDM4C | lysine demethylase 4C |
| KDM4D | lysine demethylase 4D |
| KDM4E | lysine demethylase 4E |
| KDM5A | lysine demethylase 5A |
| KDM5B | lysine demethylase 5B |
| KDM5C | lysine demethylase 5C |
| KDM5D | lysine demethylase 5D |
| KDM6A | lysine demethylase 6A |
| KDM6B | lysine demethylase 6B |
| KDM7A | lysine demethylase 7A |
| KDM8 | lysine demethylase 8 |
| OGFOD1 | 2-oxoglutarate and iron dependent oxygenase domain containing 1 |
| P3H1 | prolyl 3-hydroxylase 1 |
| P3H2 | prolyl 3-hydroxylase 2 |
| P3H3 | prolyl 3-hydroxylase 3 |
| P4HA1 | prolyl 4-hydroxylase subunit alpha 1 |
| P4HA2 | prolyl 4-hydroxylase subunit alpha 2 |
| P4HA3 | prolyl 4-hydroxylase subunit alpha 3 |
| P4HTM | prolyl 4-hydroxylase, transmembrane |
| PHF2 | PHD finger protein 2 |
| PHF8 | PHD finger protein 8 |
| PHYH | phytanoyl-CoA 2-hydroxylase |
| PHYHD1 | phytanoyl-CoA dioxygenase domain containing 1 |
| PLOD1 | procollagen-lysine,2-oxoglutarate 5-dioxygenase 1 |
| PLOD2 | procollagen-lysine,2-oxoglutarate 5-dioxygenase 2 |
| PLOD3 | procollagen-lysine,2-oxoglutarate 5-dioxygenase 3 |
| TET1 | tet methylcytosine dioxygenase 1 |
| TET2 | tet methylcytosine dioxygenase 2 |
| TET3 | tet methylcytosine dioxygenase 3 |
| TMLHE | trimethyllysine hydroxylase, epsilon |
| UTY | ubiquitously transcribed tetratricopeptide repeat containing, Y-linked |
