# Supplementary material for: Dual prognostic role of 2-oxoglutarate-dependent oxygenases in ten cancer types: implications for cell cycle regulation and cell adhesion maintenance
Source: Cancer Commun (Lond). 2019 Apr 29;39:23. doi: 10.1186/s40880-019-0369-5 (PMC6489267; doi:10.1186/s40880-019-0369-5)
Supplement: Supplementary file 3 — Additional file 3. Univariate and multivariate Cox proportional hazards analysis of risk factors associated with overall survival in multiple cancers. Univariate values of TNM stage were in accordance with our previous report utilizing TCGA datasets [4]. [file 40880_2019_369_MOESM3_ESM.docx]

| **Additional File 3. Univariate and multivariate Cox proportional hazards repression analysis of risk factors associated with overall survival in multiple cancers.** | | |
| --- | --- | --- |
|  | | |
| **Variable** | **HR (95% CI)** | ***P* value** |
| **Bladder (BLCA)** | **Univariate** | |
| Signature 1 (high *vs.* low score) | 0.662 (0.450-0.974) | 0.036 |
| Signature 2 (high *vs.* low score) | 1.459 (1.096-2.137) | 0.042 |
| TNM stage (stage II & III *vs.* I) | 1.679 (1.323-2.131) | < 0.001 |
| *PCDHA1* mutation (yes *vs.* no) | 1.649 (1.058-2.569) | 0.027 |
| *TTN* mutation (yes *vs.* no) | 1.610 (1.091-2.376) | 0.016 |
|  | **Multivariate (Signature 1)** | |
| Signature 1 (high *vs*. low score) | 0.714 (0.502-1.094) | 0.132 |
| TNM stage (stage II & III *vs.* I) | 1.636 (1.286-2.081) | < 0.001 |
|  | **Multivariate (Signature 1)** | |
| Signature 1 (high *vs.* low score) | 0.686 (0.466-0.912) | 0.047 |
| *PCDHA1* mutation (yes *vs.* no) | 1.573 (1.008-2.458) | 0.046 |
|  | **Multivariate (Signature 2)** | |
| Signature 2 (high *vs.* low score) | 1.481 (1.011-2.168) | 0.043 |
| TNM stage (stage II & III *vs.* I) | 1.685 (1.328-2.137) | < 0.001 |
|  | **Multivariate (Signature 2)** | |
| Signature 2 (low *vs.* high score) | 1.411 (1.062-2.070) | 0.048 |
| *PCDHA1* mutation (yes *vs.* no) | 1.584 (1.015-2.472) | 0.043 |
|  |  |  |
|  |  |  |
| **Cervical (CESC)** | **Univariate** | |
| Signature 2 (high *vs.* low score) | 1.972 (1.003-3.877) | 0.045 |
| TNM stage (stage II & III *vs.* I) | 1.017 (0.695-1.487) | 0.930 |
| *PCDHA1* mutation (yes *vs.*no) | 1.364 (0.681-2.729) | 0.380 |
| *PIK3CA* mutation (yes *vs.* no) | 1.906 (0.955-3.804) | 0.067 |
|  | **Multivariate (Signature 2)** | |
| Signature 2 (high *vs.* low score) | 2.010 (1.011-3.996) | 0.046 |
| TNM stage (stage II & III *vs.* I) | 0.942 (0.639-1.390) | 0.760 |
|  |  |  |
|  |  |  |
| **Head and neck (HNSC)** | **Univariate** | |
| Signature 2 (high *vs.* low score) | 1.479 (1.056-2.072) | 0.023 |
| TNM stage (stage II & III *vs.* I) | 1.466 (1.188-1.808) | < 0.001 |
|  | **Multivariate (Signature 2)** | |
| Signature 2 (high *vs.* low score) | 1.344 (0.956-1.890) | 0.089 |
| TNM stage (stage II & III *vs.* I) | 1.426 (1.155-1.761) | < 0.001 |
|  |  |  |
|  |  |  |
| **Renal clear cell (KIRC)** | **Univariate** | |
| Signature 2 (high *vs.* low score) | 1.483 (1.096-2.007) | 0.011 |
| TNM stage (stage II & III *vs.* I) | 1.870 (1.641-2.132) | < 0.001 |
| *MUC4* mutation (yes *vs.* no) | 0.570 (0.370-0.880) | 0.012 |
| *PBRM1* mutation (yes *vs.* no) | 0.771 (0.508-1.172) | 0.220 |
| *VHL* mutation (yes *vs.* no) | 1.056 (0.780-1.430) | 0.720 |
|  | **Multivariate (Signature 2)** | |
| Signature 2 (high *vs.* low score) | 1.197 (0.881-1.627) | 0.250 |
| TNM stage (stage II & III *vs.* I) | 1.847 (1.618-2.109) | < 0.001 |
|  | **Multivariate (Signature 2)** | |
| Signature 2 (high *vs.* low score) | 1.520 (1.123-2.056) | 0.007 |
| *MUC4* mutation (yes *vs.* no) | 0.554 (0.359-0.854) | 0.008 |
|  |  |  |
|  |  |  |
| **Renal papillary cell (KIRP)** | **Univariate** | |
| Signature 1 (high *vs.* low score) | 0.370 (0.157-0.871) | 0.023 |
| Signature 2 (high *vs.* low score) | 3.862 (1.565-9.526) | 0.003 |
| TNM stage (stage II & III vs. I) | 2.710 (1.893-3.878) | < 0.001 |
|  | **Multivariate (Signature 1)** | |
| Signature 1 (high *vs.* low score) | 0.461 (0.194-0.981) | 0.048 |
| TNM stage (stage II & III *vs.* I) | 2.621 (1.821-3.772) | < 0.001 |
|  | **Multivariate (Signature 2)** | |
| Signature 2 (high *vs.* low score) | 3.057 (1.232-7.584) | 0.016 |
| TNM stage (stage II & III *vs.* I) | 2.616 (1.805-3.790) | < 0.001 |
|  |  |  |
|  |  |  |
| **Liver #1 (LIHC)** | **Univariate** | |
| Signature 1 (high *vs.* low score) | 0.656 (0.424-0.915) | 0.048 |
| TNM stage (stage II & III *vs.* I) | 2.000 (1.533-2.608) | < 0.001 |
|  | **Multivariate (Signature 1)** | |
| Signature 1 (high *vs.* low score) | 0.717 (0.464-1.108) | 0.130 |
| TNM stage (stage II & III *vs.* I) | 1.999 (1.570-2.546) | < 0.001 |
|  |  |  |
|  |  |  |
| **Liver #2 (LIRI-JP)** | **Univariate** | |
| Signature 1 (high *vs.* low score) | 0.490 (0.259-0.938) | 0.031 |
| Signature 2 (high *vs.* low score) | 5.271 (2.429-11.44) | < 0.001 |
| Tumour size (> 3cm *vs.* < 3cm) | 2.415 (1.263-4.615) | 0.008 |
| TNM stage (stage II & III *vs.* I) | 2.267 (1.556-3.304) | < 0.001 |
|  | **Multivariate (Signature 1)** | |
| Signature 1 (high *vs.* low score) | 0.541 (0.283-0.904) | 0.043 |
| Tumour size (> 3cm *vs.* < 3cm) | 1.003 (0.997-1.009) | 0.380 |
| TNM stage (stage II & III *vs.* I) | 2.065 (1.374-3.104) | < 0.001 |
|  | **Multivariate (Signature 2)** | |
| Signature 2 (high *vs.* low score) | 4.539 (2.055-10.029) | < 0.001 |
| Tumour size (> 3 cm *vs.* < 3 cm) | 1.001 (0.994-1.008) | 0.780 |
| TNM stage (stage II & III *vs.* I) | 1.926 (1.296-2.862) | < 0.001 |
|  |  |  |
|  |  |  |
| **Liver #3 (GSE14520)** | **Univariate** | |
| Signature 2 (high *vs.* low score) | 2.285 (1.458-3.580) | < 0.001 |
| Tumour size (> 5cm *vs.* < 5cm) | 2.159 (1.406-3.316) | < 0.001 |
| Cirrhosis (yes *vs.* no) | 4.665 (1.147-18.97) | 0.031 |
| TNM stage (stage II & III *vs.* I) | 2.260 (1.706-2.994) | < 0.001 |
| BCLC stage (stage B & C *vs.* A & 0) | 2.181 (1.722-2.762) | < 0.001 |
| AFP (> 300 ng/mL *vs.* < 300 ng/mL) | 1.606 (1.049-2.46) | 0.029 |
|  | **Multivariate (Signature 2)** | |
| Signature 2 (high *vs.* low score) | 2.012 (1.267-3.195) | 0.003 |
| Tumour size (> 5 cm *vs.* < 5 cm) | 0.983 (0.589-1.642) | 0.940 |
| Cirrhosis (yes *vs.* no) | 3.187 (0.762-13.335) | 0.110 |
| TNM stage (stage II & III *vs*. I) | 1.346 (0.914-1.980) | 0.130 |
| BCLC stage (stage B & C *vs.* A & 0) | 1.873 (1.332-2.634) | < 0.001 |
|  |  |  |
|  |  |  |
| **Lung (LUAD)** | **Univariate** | |
| Signature 1 (high *vs.* low score) | 0.625 (0.443-0.879) | 0.007 |
| Signature 2 (high *vs.* low score) | 1.562 (1.116-2.188) | 0.009 |
| TNM stage (stage II & III *vs.* I) | 1.597 (1.364-1.870) | < 0.001 |
|  | **Multivariate (Signature 1)** | |
| Signature 1 (high *vs.* low score) | 0.715 (0.505-0.913) | 0.039 |
| TNM stage (stage II & III *vs.* I) | 1.553 (1.324-1.822) | < 0.001 |
|  | **Multivariate (Signature 2)** | |
| Signature 2 (high *vs.* low score) | 1.496 (1.067-2.097) | 0.019 |
| TNM stage (stage II & III *vs.* I) | 1.587 (1.353-1.861) | < 0.001 |
|  |  |  |
|  |  |  |
| **Pancreas (PAAD)** | **Univariate** | |
| Signature 1 (high *vs.* low score) | 0.454 (0.278-0.741) | 0.002 |
| Signature 2 (high *vs.* low score) | 1.969 (1.217-3.186) | 0.006 |
| TNM stage (stage II & III *vs.* I) | 1.339 (0.897-1.998) | 0.153 |
|  | **Multivariate (Signature 1)** | |
| Signature 1 (high *vs.* low score) | 0.472 (0.284-0.783) | 0.004 |
| TNM stage (stage II & III *vs.* I) | 1.150 (0.722-1.832) | 0.560 |
|  | **Multivariate (Signature 2)** | |
| Signature 2 (high *vs.* low score) | 1.888 (1.157-3.082) | 0.011 |
| TNM stage (stage II & III *vs.* I) | 1.205 (0.785-1.851) | 0.394 |
|  |  |  |
|  |  |  |
| **Stomach (STAD)** | **Univariate** | |
| Signature 2 (high *vs.* low score) | 1.725 (1.142-2.605) | 0.009 |
| TNM stage (stage II & III *vs.* I) | 1.372 (1.067-1.765) | 0.013 |
| *PCDHA1* mutation (yes *vs.*no) | 1.525 (1.007-2.307) | 0.046 |
| *PCDHA2* mutation (yes *vs.* no) | 1.604 (1.061-2.427) | 0.025 |
| *TP53* mutation (yes *vs.* no) | 1.296 (0.853-1.967) | 0.220 |
| *TTN* mutation (yes *vs.* no) | 1.115 (0.740-1.682) | 0.600 |
|  | **Multivariate (Signature 2)** | |
| Signature 2 (high *vs.* low score) | 1.800 (1.184-2.737) | 0.006 |
| TNM stage (stage II & III *vs.* I) | 1.464 (1.130-1.898) | 0.004 |
| *PCDHA1* mutation (yes *vs.* no) | 1.501 (0.988-2.281) | 0.056 |
| *PCDHA2* mutation (yes *vs.* no) | 1.558 (1.026-2.365) | 0.037 |
|  |  |  |
|  |  |  |
| **Endometrial (UCEC)** | **Univariate** | |
| Signature 1 (high *vs.* low score) | 0.401 (0.229-0.702) | 0.002 |
| TNM stage (stage II & III *vs.* I) | 1.802 (1.431-2.269) | < 0.001 |
| *PCDHA1* mutation (yes *vs.* no) | 0.516 (0.272-0.978) | 0.042 |
| *PIK3CA* mutation (yes *vs.* no) | 0.362 (0.190-0.689) | 0.002 |
| *PIK3R1* mutation (yes *vs.* no) | 1.180 (0.645-2.158) | 0.592 |
| *PTEN* mutation (yes *vs.* no) | 0.427 (0.234-0.781) | 0.006 |
| *TP53* mutation (yes *vs.* no) | 1.780 (1.025-3.090) | 0.041 |
|  | **Multivariate (Signature 1)** | |
| Signature 1 (high *vs.* low score) | 0.519 (0.293-0.920) | 0.024 |
| TNM stage (stage II & III *vs.* I) | 1.589 (1.245-2.028) | < 0.001 |
| *PCDHA1* mutation (yes *vs.* no) | 0.803 (0.376-1.712) | 0.560 |
| *PIK3CA* mutation (yes *vs.* no) | 0.428 (0.216-0.848) | 0.015 |
| *PTEN* mutation (yes *vs.* no) | 0.904 (0.427-1.910) | 0.790 |
| *TP53* mutation (yes *vs.* no) | 1.567 (0.867-2.832) | 0.130 |

HR, hazard ratio; CI, confidence interval; BCLC stageing, Barcelona clinic liver cancer stageing; AFP, alpha-Fetoprotein.
