# Supplementary figures and images for: Dual prognostic role of 2-oxoglutarate-dependent oxygenases in ten cancer types: implications for cell cycle regulation and cell adhesion maintenance
Source: Cancer Commun (Lond). 2019 Apr 29;39:23. doi: 10.1186/s40880-019-0369-5 (PMC6489267; doi:10.1186/s40880-019-0369-5)

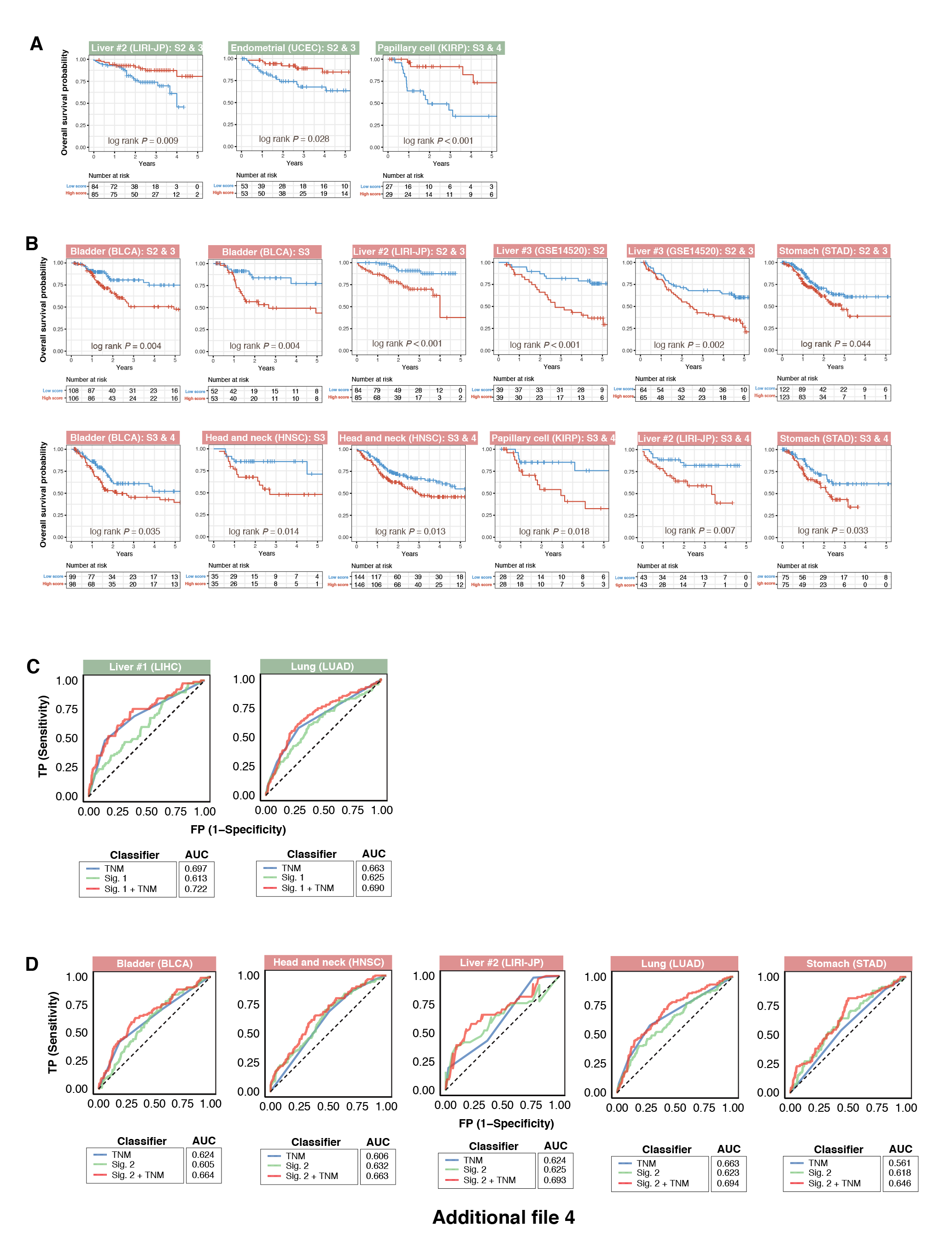

Supplement: Supplementary file 4 — Additional file 4. Additional tumor subgroup analyses and evaluation of prognosis predictive performance of gene signatures across different malignant grades. Kaplan–Meier plots show independence of (A) signature 1 (green panels) and (B) signature 2 (red panels) over current TNM staging system in predicting prognosis in different cancer cohorts. Patients were sub-grouped according to TNM stages and further stratified using either signature 1 or signature 2 scores. Both signatures successfully identified high-risk patients in different TNM stages. P values were calculated from the log-rank test. Analysis of specificity and sensitivity of (C) signature 1 (green panels) and (D) signature 2 (red panels) in predicting prognosis in different cancer cohorts using receiver operating characteristic (ROC) curves. Plots depict comparison of ROC curves of signature 1 or 2 and clinical TNM staging. Both signatures demonstrated incremental values over current TNM staging system. AUC: area under the curve. TNM: tumor, node, metastasis staging. Liver #1 = LIHC cohort; Liver #2 = LIRI-JP cohort and Liver #3 = GSE14520 cohort (Additional file 1). [file 40880_2019_369_MOESM4_ESM.tif]

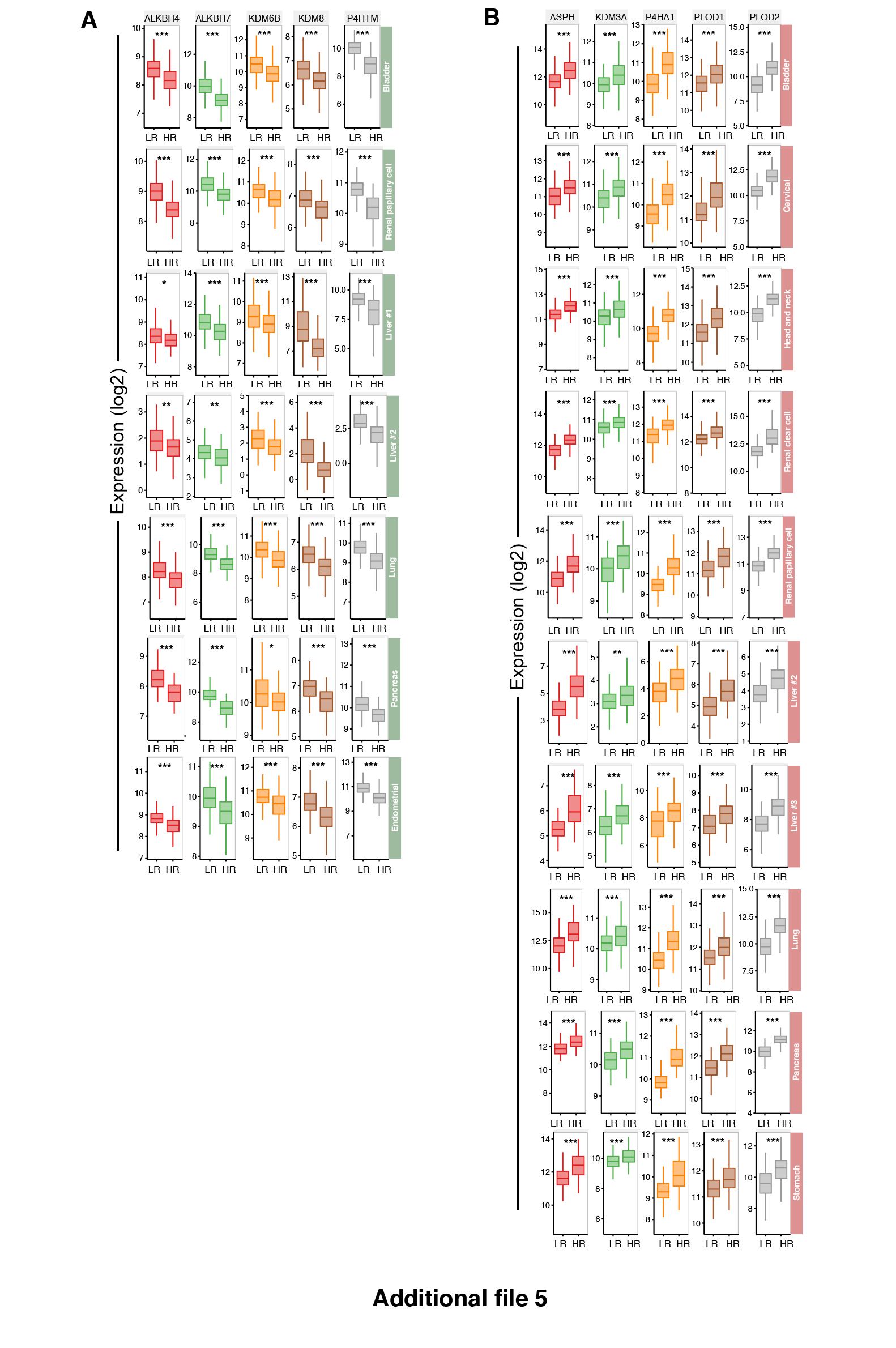

Supplement: Supplementary file 5 — Additional file 5. Distribution of expression of signature genes in low- and high-risk patients. (A) signature 1 (green panels) and (B) signature 2 (red panels). Patients were median-stratified into low- and high-risk groups based on mean expression scores of signature genes. Box plots depict expression distribution of each of the 5 genes in both signatures in these two patient groups. (A) Since signature 1 is a marker of good prognosis, high-risk patients show significantly lower expression of individual signature genes. (B) In contrast, signature 2 is a marker of poor prognosis, hence high-risk patients show significantly higher expression of individual signature genes. Nonparametric Mann–Whitney–Wilcoxon tests were used to compare low- and high-risk patients. Asterisks represent significant P values: * < 0.01, ** < 0.001 and *** < 0.0001. LR = low risk. HR = high risk. [file 40880_2019_369_MOESM5_ESM.tif]

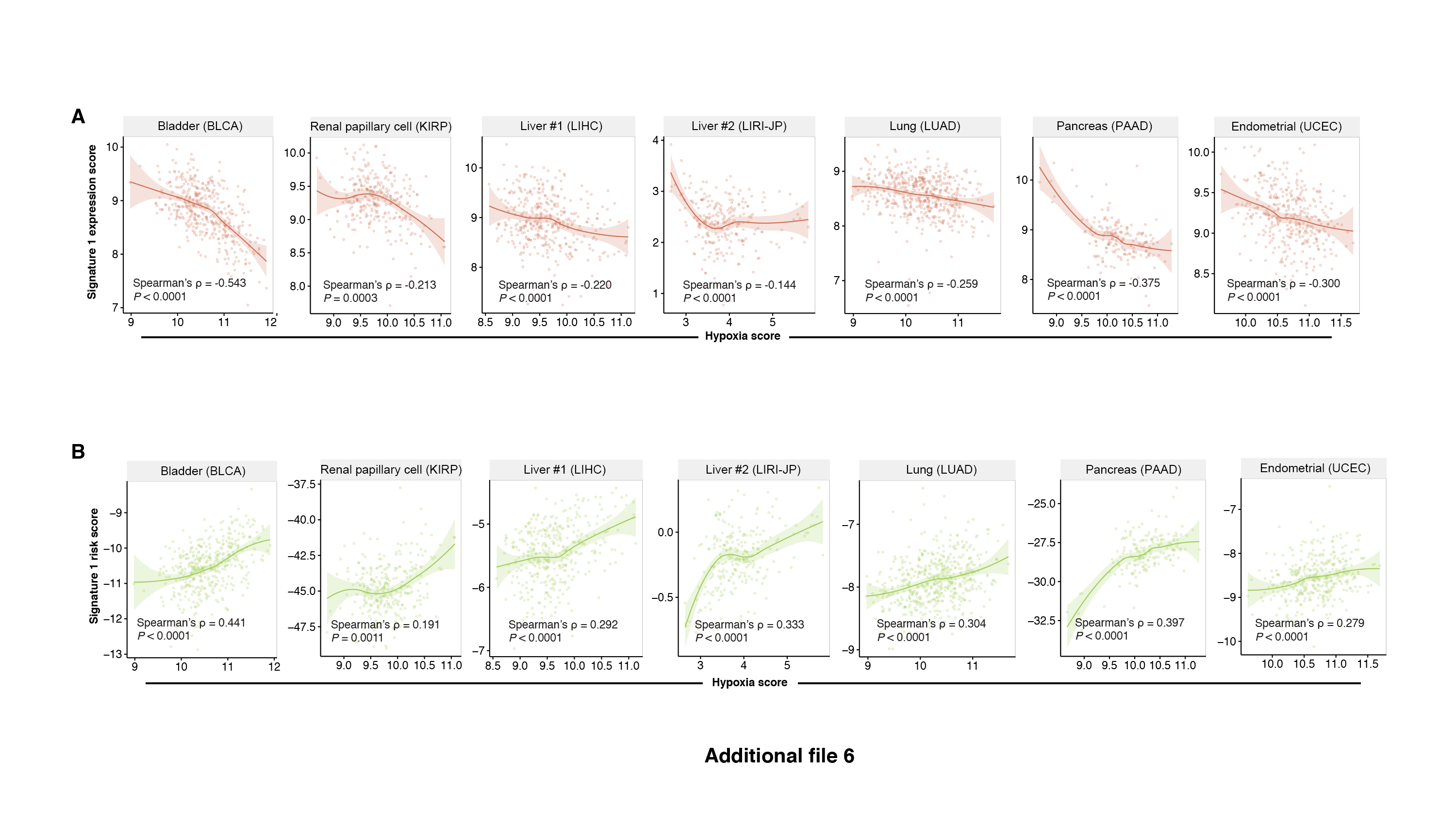

Supplement: Supplementary file 6 — Additional file 6. Correlation of patients’ risk scores derived from signature 1 with tumor hypoxia. (A) Significant negative correlation between signature 1 expression scores and tumor hypoxia. (B) Significant positive correlation between signature 1 risk scores and tumor hypoxia. Calculations of expression scores, risk scores, and hypoxia scores are explained in the methods. Liver #1 = LIHC cohort and Liver #2 = LIRI-JP cohort. [file 40880_2019_369_MOESM6_ESM.tif]

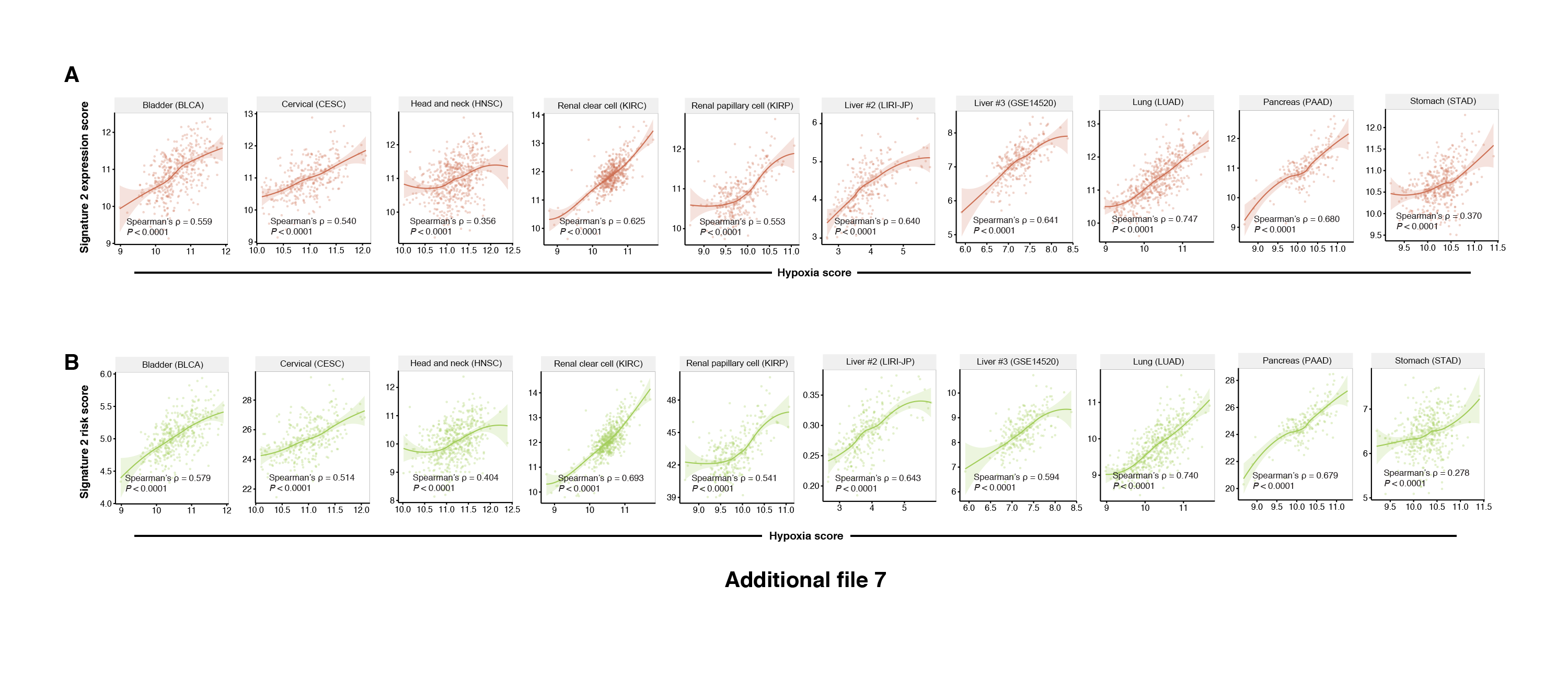

Supplement: Supplementary file 7 — Additional file 7. Correlation of patients’ risk scores derived from signature 2 with tumor hypoxia. (A) Significant positive correlation between signature 2 expression scores and tumor hypoxia. (B) Significant positive correlation between signature 2 risk scores and tumor hypoxia. Calculations of expression scores, risk scores and hypoxia scores are explained in the methods. Liver #2 = LIRI-JP cohort and Liver #3 = GSE14520 cohort. [file 40880_2019_369_MOESM7_ESM.tif]
