# Supplementary material for: Dual prognostic role of 2-oxoglutarate-dependent oxygenases in ten cancer types: implications for cell cycle regulation and cell adhesion maintenance
Source: Cancer Commun (Lond). 2019 Apr 29;39:23. doi: 10.1186/s40880-019-0369-5 (PMC6489267; doi:10.1186/s40880-019-0369-5)
Supplement: Supplementary file 8 — Additional file 8. Differentially expressed genes between KDM8-high and -low groups in the liver cancer cohort (LIHC). [file 40880_2019_369_MOESM8_ESM.docx]

| **Additional file 8. Differentially expressed genes between KDM8-high and -low groups in liver cancer cohort (LIHC)** | | | |
| --- | --- | --- | --- |
|  |  |  |  |
| **Entrez ID** | **Gene symbol** | **Log2 fold change** | ***P* value** |
| 1548 | CYP2A6 | 4.266217426 | 0.001070647 |
| 1409 | CRYAA | 3.782386136 | 0.000184121 |
| 1576 | CYP3A4 | 3.698152017 | 0.004124437 |
| 1582 | CYP8B1 | 3.644639707 | 0.000565742 |
| 255167 | LINC01018 | 3.620703779 | 0.001817311 |
| 143941 | TTC36 | 3.602776338 | 0.000293049 |
| 51179 | HAO2 | 3.554412019 | 0.000892977 |
| 64388 | GREM2 | 3.515557145 | 0.001483312 |
| 1549 | CYP2A7 | 3.471653994 | 0.009032202 |
| 388503 | C3P1 | 3.446255927 | 0.000123142 |
| 2642 | GCGR | 3.442241631 | 0.004731164 |
| 6718 | AKR1D1 | 3.309733658 | 0.000704509 |
| 2645 | GCK | 3.286380385 | 0.003725235 |
| 173 | AFM | 3.274019175 | 0.000487471 |
| 28234 | SLCO1B3 | 3.200697658 | 0.013857647 |
| 6554 | SLC10A1 | 3.084096620 | 0.002198269 |
| 2674 | GFRA1 | 3.066645866 | 0.000515341 |
| 252995 | FNDC5 | 3.049912992 | 0.000542378 |
| 33 | ACADL | 3.043110654 | 0.000960604 |
| 10877 | CFHR4 | 3.023986393 | 0.000576045 |
| 84879 | MFSD2A | 3.003142647 | 0.000328791 |
| 635 | BHMT | 2.973505474 | 0.001225789 |
| 127 | ADH4 | 2.925047346 | 0.006456936 |
| 1581 | CYP7A1 | 2.918451657 | 0.007607397 |
| 10249 | GLYAT | 2.890068540 | 0.005452309 |
| 7069 | THRSP | 2.878250330 | 0.011128485 |
| 10864 | SLC22A7 | 2.829437600 | 0.002915624 |
| 6694 | SPP2 | 2.799569821 | 0.004013825 |
| 266629 | SEC14L3 | 2.785909232 | 0.000029984 |
| 6898 | TAT | 2.764924205 | 0.002283852 |
| 1610 | DAO | 2.700033063 | 0.001676755 |
| 8647 | ABCB11 | 2.688533260 | 0.011722721 |
| 5244 | ABCB4 | 2.687798988 | 0.000106696 |
| 10 | NAT2 | 2.679791071 | 0.001921913 |
| 83597 | RTP3 | 2.672686193 | 0.001081366 |
| 189 | AGXT | 2.600530207 | 0.000591273 |
| 4311 | MME | 2.597010495 | 0.028115017 |
| 171389 | NLRP6 | 2.576924698 | 0.000017337 |
| 1600 | DAB1 | 2.558491234 | 0.000062006 |
| 58503 | OPRPN | 2.531857402 | 0.004723015 |
| 284541 | CYP4A22 | 2.526945213 | 0.002622958 |
| 3626 | INHBC | 2.514424591 | 0.000155015 |
| 116519 | APOA5 | 2.512852835 | 0.0008519 |
| 2158 | F9 | 2.505159911 | 0.011036784 |
| 200931 | SLC51A | 2.497961255 | 0.003342533 |
| 2998 | GYS2 | 2.494680586 | 0.011620599 |
| 202299 | N/A | 2.488584103 | 0.018415021 |
| 92292 | GLYATL1 | 2.484104891 | 0.000030912 |
| 51302 | CYP39A1 | 2.452502730 | 0.005432105 |
| 29947 | DNMT3L | 2.447810506 | 0.001451150 |
| 7038 | TG | 2.429091048 | 0.001987957 |
| 150280 | HORMAD2 | 2.428598050 | 0.000156247 |
| 51733 | UPB1 | 2.427539750 | 0.002804778 |
| 462 | SERPINC1 | 2.423306661 | 0.007685312 |
| 26998 | FETUB | 2.423205357 | 0.006804976 |
| 345 | APOC3 | 2.414959144 | 0.001946819 |
| 11199 | ANXA10 | 2.410635640 | 0.000373832 |
| 2892 | GRIA3 | 2.376439020 | 0.006892948 |
| 123876 | ACSM2A | 2.375149513 | 0.001677471 |
| 8529 | CYP4F2 | 2.356139541 | 0.003590649 |
| 1373 | CPS1 | 2.347456038 | 0.031293508 |
| 1564 | N/A | 2.344177514 | 0.001167081 |
| 6580 | SLC22A1 | 2.327872835 | 0.027051449 |
| 729 | C6 | 2.324475916 | 0.000002066 |
| 1558 | CYP2C8 | 2.317696049 | 0.001559205 |
| 5444 | PON1 | 2.316342538 | 0.001985561 |
| 1579 | CYP4A11 | 2.315220723 | 0.000471545 |
| 157310 | PEBP4 | 2.296432128 | 0.003869673 |
| 57733 | GBA3 | 2.291043867 | 0.003399187 |
| 64902 | AGXT2 | 2.279171841 | 0.006002827 |
| 816 | CAMK2B | 2.274354004 | 0.014691834 |
| 29943 | PADI1 | 2.265481392 | 0.018050291 |
| 23086 | EXPH5 | 2.258753783 | 0.000358240 |
| 7545 | ZIC1 | 2.242916061 | 0.009744838 |
| 8630 | HSD17B6 | 2.234080306 | 0.004389488 |
| 10878 | CFHR3 | 2.229794129 | 0.017950652 |
| 23316 | CUX2 | 2.219746125 | 0.018957676 |
| 348158 | ACSM2B | 2.216326931 | 0.001903964 |
| 57451 | TENM2 | 2.215244511 | 0.019270350 |
| 9077 | DIRAS3 | 2.202948942 | 0.000237404 |
| 81494 | CFHR5 | 2.197465122 | 0.040225546 |
| 8856 | NR1I2 | 2.179939607 | 0.016093996 |
| 229 | ALDOB | 2.179066312 | 0.010830097 |
| 1557 | CYP2C19 | 2.179047891 | 0.000446408 |
| 27232 | GNMT | 2.178235399 | 0.012158931 |
| 22977 | AKR7A3 | 2.175902379 | 0.009147347 |
| 731 | C8A | 2.169356533 | 0.001001301 |
| 6514 | SLC2A2 | 2.162013000 | 0.013804129 |
| 3950 | LECT2 | 2.140551880 | 0.046697952 |
| 63982 | ANO3 | 2.136028037 | 0.001540142 |
| 1553 | CYP2A13 | 2.132585759 | 0.016606607 |
| 3053 | SERPIND1 | 2.113853805 | 0.008433887 |
| 83854 | ANGPTL6 | 2.113590149 | 0.000128373 |
| 121643 | FOXN4 | 2.099987483 | 0.019830535 |
| 3250 | HPR | 2.099986491 | 0.014752115 |
| 124 | ADH1A | 2.094269712 | 0.004377302 |
| 366 | AQP9 | 2.092012283 | 0.009955353 |
| 3483 | IGFALS | 2.083719247 | 0.014959325 |
| 145264 | SERPINA12 | 2.079006118 | 0.001337341 |
| 83729 | INHBE | 2.073728990 | 0.001235156 |
| 2775 | GNAO1 | 2.073549717 | 0.001254919 |
| 404037 | HAPLN4 | 2.072589055 | 0.000548267 |
| 8608 | RDH16 | 2.072137893 | 0.011061050 |
| 2822 | GPLD1 | 2.071295157 | 0.013408377 |
| 1565 | CYP2D6 | 2.069534836 | 0.009360876 |
| 6360 | CCL16 | 2.068833248 | 0.017718212 |
| 10747 | MASP2 | 2.067838126 | 0.016925366 |
| 5789 | PTPRD | 2.065981194 | 0.015065062 |
| 116842 | LEAP2 | 2.062777241 | 0.002171084 |
| 653808 | ZG16 | 2.062233472 | 0.020038666 |
| 10599 | SLCO1B1 | 2.052246346 | 0.008510799 |
| 5207 | PFKFB1 | 2.047236592 | 0.003951254 |
| 26291 | FGF21 | 2.043516335 | 0.033162519 |
| 53345 | TM6SF2 | 2.037578069 | 0.001128643 |
| 80168 | MOGAT2 | 2.031577131 | 0.011018668 |
| 2099 | ESR1 | 2.031557510 | 0.002132853 |
| 56134 | PCDHAC2 | 2.030624527 | 0.007205624 |
| 283537 | SLC46A3 | 2.024509926 | 0.000149799 |
| 9970 | NR1I3 | 2.021507726 | 0.008723175 |
| 2161 | F12 | 2.021505846 | 0.008103316 |
| 280 | AMY2B | 2.020923782 | 0.000001966 |
| 148741 | ANKRD35 | 2.018621863 | 0.007869293 |
| 429 | ASCL1 | 2.013026287 | 0.014483865 |
| 346 | APOC4 | 2.012881975 | 0.030557686 |
| 360200 | TMPRSS9 | 2.010677453 | 0.011300575 |
| 134111 | UBE2QL1 | 2.007580313 | 0.006600179 |
| 1828 | DSG1 | 2.003596641 | 0.025063577 |
| 2165 | F13B | 2.000652263 | 0.009233682 |
| 3827 | KNG1 | 1.997414991 | 0.002147747 |
| 64850 | ETNPPL | 1.992437952 | 0.028921785 |
| 23541 | SEC14L2 | 1.988841343 | 0.000171278 |
| 387601 | SLC22A25 | 1.988373871 | 0.002571215 |
| 392636 | AGMO | 1.982612445 | 0.007127166 |
| 1555 | CYP2B6 | 1.974663087 | 0.004152261 |
| 2690 | GHR | 1.971209960 | 0.000034915 |
| 388646 | GBP7 | 1.969293624 | 0.045293980 |
| 5340 | PLG | 1.968029917 | 0.006855863 |
| 53841 | CDHR5 | 1.965182625 | 0.012015737 |
| 221223 | CES5A | 1.963985876 | 0.002671821 |
| 6291 | SAA4 | 1.963317102 | 0.016743223 |
| 151126 | ZNF385B | 1.959984183 | 0.008367854 |
| 126 | ADH1C | 1.955094511 | 0.045514905 |
| 151295 | SLC23A3 | 1.952474699 | 0.007351873 |
| 2938 | GSTA1 | 1.948694820 | 0.043904746 |
| 114571 | SLC22A9 | 1.939303807 | 0.004427560 |
| 3263 | HPX | 1.938423562 | 0.008971805 |
| 335 | APOA1 | 1.937796701 | 0.020326550 |
| 4018 | LPA | 1.935111258 | 0.004113881 |
| 23584 | VSIG2 | 1.930836804 | 0.030319734 |
| 3818 | KLKB1 | 1.930832589 | 0.000004202 |
| 159963 | SLC5A12 | 1.926473098 | 0.006415145 |
| 64816 | CYP3A43 | 1.918812267 | 0.001628690 |
| 114770 | PGLYRP2 | 1.918751789 | 0.024852669 |
| 26 | AOC1 | 1.918632919 | 0.010663639 |
| 51268 | PIPOX | 1.916083330 | 0.002293964 |
| 339398 | LINGO4 | 1.914251522 | 0.002619155 |
| 389643 | NUGGC | 1.910340137 | 0.011092759 |
| 9388 | LIPG | 1.909470603 | 0.000104456 |
| 122664 | TPPP2 | 1.908114969 | 0.001873702 |
| 3240 | HP | 1.906797774 | 0.022811484 |
| 54988 | ACSM5 | 1.903249856 | 0.015671305 |
| 6716 | SRD5A2 | 1.902798702 | 0.012995147 |
| 7399 | USH2A | 1.901861001 | 0.006155153 |
| 6822 | SULT2A1 | 1.896825407 | 0.027345031 |
| 389434 | IYD | 1.889670162 | 0.001229500 |
| 2160 | F11 | 1.886585889 | 0.001108506 |
| 10998 | SLC27A5 | 1.886493737 | 0.004258579 |
| 3080 | CFHR2 | 1.872150388 | 0.016205216 |
| 387778 | SPDYC | 1.870768999 | 0.001686804 |
| 4153 | MBL2 | 1.865182539 | 0.020969296 |
| 6751 | SSTR1 | 1.853287797 | 0.010644500 |
| 256394 | SERPINA11 | 1.853130059 | 0.027811459 |
| 164656 | TMPRSS6 | 1.848365981 | 0.004707227 |
| 55908 | ANGPTL8 | 1.843952506 | 0.021207540 |
| 1 | A1BG | 1.841239211 | 0.019645158 |
| 590 | BCHE | 1.838711089 | 0.047180947 |
| 201651 | AADACP1 | 1.829664266 | 0.002100734 |
| 7276 | TTR | 1.826134518 | 0.022078856 |
| 55224 | ETNK2 | 1.819980809 | 0.005221762 |
| 554235 | ASPDH | 1.814205909 | 0.031954450 |
| 1036 | CDO1 | 1.812630613 | 0.005704286 |
| 654790 | PCP4L1 | 1.812336409 | 0.015486964 |
| 1109 | AKR1C4 | 1.798881786 | 0.024654002 |
| 7365 | UGT2B10 | 1.797549880 | 0.006054442 |
| 79831 | KDM8 | 1.797348411 | 5.14958E-06 |
| 83875 | BCO2 | 1.790680930 | 0.002815352 |
| 1592 | CYP26A1 | 1.790051825 | 0.008741061 |
| 8858 | PROZ | 1.789316028 | 0.012357988 |
| 2894 | GRID1 | 1.784642645 | 0.038421082 |
| 6529 | SLC6A1 | 1.777487014 | 0.002787926 |
| 135138 | PACRG | 1.776624146 | 0.016016102 |
| 85480 | TSLP | 1.772797812 | 0.008926007 |
| 346606 | MOGAT3 | 1.772604380 | 0.010890367 |
| 6540 | SLC6A13 | 1.772129375 | 0.006988695 |
| 6539 | SLC6A12 | 1.760580601 | 0.004598944 |
| 50486 | G0S2 | 1.759248375 | 0.002818565 |
| 23491 | CES3 | 1.758411582 | 0.005206800 |
| 91947 | ARRDC4 | 1.754857186 | 0.000627529 |
| 10586 | MAB21L2 | 1.750944770 | 0.027107321 |
| 400830 | DEFB132 | 1.746677951 | 0.019190447 |
| 276 | AMY1A | 1.745153531 | 0.000151813 |
| 57549 | IGSF9 | 1.740577196 | 0.047837840 |
| 246778 | IL27 | 1.738596308 | 0.002396079 |
| 51458 | RHCG | 1.732390615 | 0.046747665 |
| 552 | AVPR1A | 1.729890419 | 0.044513270 |
| 84107 | ZIC4 | 1.729712360 | 0.028360710 |
| 145837 | N/A | 1.728976489 | 0.047849345 |
| 148738 | HFE2 | 1.722431132 | 0.048661379 |
| 7306 | TYRP1 | 1.713379317 | 0.012134900 |
| 2041 | EPHA1 | 1.698195127 | 0.028153750 |
| 1559 | CYP2C9 | 1.692305393 | 0.017770237 |
| 55753 | OGDHL | 1.687834165 | 0.014810533 |
| 1238 | ACKR2 | 1.686749210 | 0.001267128 |
| 1101 | CHAD | 1.681941072 | 0.000812831 |
| 1962 | EHHADH | 1.679240877 | 0.001395897 |
| 93145 | OLFM2 | 1.678413754 | 0.000480129 |
| 22843 | PPM1E | 1.676015108 | 0.030930921 |
| 1370 | CPN2 | 1.669817786 | 0.007900849 |
| 3948 | LDHC | 1.669309207 | 0.017403664 |
| 6579 | SLCO1A2 | 1.668373987 | 0.038648928 |
| 63951 | DMRTA1 | 1.667196203 | 0.016829527 |
| 84866 | TMEM25 | 1.663207975 | 0.003196440 |
| 81029 | WNT5B | 1.661039019 | 0.010439984 |
| 90523 | MLIP | 1.659916179 | 0.012463271 |
| 732 | C8B | 1.652146764 | 0.006573558 |
| 57512 | GPR158 | 1.642390106 | 0.040030886 |
| 23632 | CA14 | 1.641714687 | 0.018466979 |
| 284904 | SEC14L4 | 1.640343954 | 0.041929967 |
| 80069 | LINC00574 | 1.637356735 | 0.000202390 |
| 91703 | ACY3 | 1.634570238 | 0.006099373 |
| 10991 | SLC38A3 | 1.632561777 | 0.016964618 |
| 2328 | FMO3 | 1.624156072 | 0.007172622 |
| 283 | ANG | 1.614849520 | 0.000565227 |
| 123872 | DNAAF1 | 1.610650547 | 0.022067570 |
| 3700 | ITIH4 | 1.610072628 | 0.003507918 |
| 57529 | RTL9 | 1.608977194 | 0.014122214 |
| 367 | AR | 1.606428173 | 0.011577899 |
| 389668 | XKR9 | 1.605973821 | 0.022433951 |
| 2538 | G6PC | 1.603549774 | 0.023203687 |
| 2180 | ACSL1 | 1.602940285 | 0.001107126 |
| 125 | ADH1B | 1.602850062 | 0.039904227 |
| 54435 | N/A | 1.598461408 | 0.028224472 |
| 64240 | ABCG5 | 1.587709404 | 0.014126658 |
| 1562 | CYP2C18 | 1.584036942 | 0.018942124 |
| 202151 | RANBP3L | 1.582539278 | 0.032435377 |
| 23460 | ABCA6 | 1.581982582 | 0.011053131 |
| 26084 | ARHGEF26 | 1.577161349 | 0.008229287 |
| 80059 | LRRTM4 | 1.575267801 | 0.004757661 |
| 7036 | TFR2 | 1.572243500 | 0.019942960 |
| 563 | AZGP1 | 1.570740021 | 0.006373881 |
| 10350 | ABCA9 | 1.569171764 | 0.000594989 |
| 5104 | SERPINA5 | 1.567646130 | 0.013542499 |
| 54363 | HAO1 | 1.561769397 | 0.028920354 |
| 84649 | DGAT2 | 1.561613212 | 0.006838670 |
| 648740 | N/A | 1.560120967 | 0.000465744 |
| 8110 | DPF3 | 1.558182368 | 0.001659700 |
| 344 | APOC2 | 1.553824300 | 0.045210088 |
| 80303 | EFHD1 | 1.551792486 | 0.004610191 |
| 26232 | FBXO2 | 1.545764839 | 0.014934684 |
| 161247 | FITM1 | 1.543109731 | 0.003448180 |
| 8991 | SELENBP1 | 1.540862076 | 0.001509125 |
| 145645 | TERB2 | 1.539483171 | 0.005321637 |
| 9832 | JAKMIP2 | 1.536765947 | 0.005393749 |
| 26577 | PCOLCE2 | 1.533702026 | 0.048591081 |
| 7018 | TF | 1.531483027 | 0.021964166 |
| 10841 | FTCD | 1.522763286 | 0.029823669 |
| 64173 | N/A | 1.521882145 | 6.26393E-05 |
| 64577 | ALDH8A1 | 1.520105598 | 0.020042599 |
| 7433 | VIPR1 | 1.517951622 | 0.009486735 |
| 283848 | CES4A | 1.505301997 | 0.020333399 |
| 134526 | ACOT12 | 1.501796858 | 0.041294440 |
| 83758 | RBP5 | 1.499639144 | 0.008899611 |
| 9848 | MFAP3L | 1.497362893 | 0.003120189 |
| 4143 | MAT1A | 1.496540752 | 0.021435203 |
| 11001 | SLC27A2 | 1.487431618 | 0.025563174 |
| 3931 | LCAT | 1.486153855 | 0.009723114 |
| 875 | CBS | 1.484246243 | 0.009582261 |
| 7498 | XDH | 1.483324178 | 0.024182197 |
| 339965 | CCDC158 | 1.479959669 | 0.001183926 |
| 3479 | IGF1 | 1.475203183 | 0.013132302 |
| 5648 | MASP1 | 1.473335053 | 0.003850723 |
| 1621 | DBH | 1.468504375 | 0.025107006 |
| 2564 | GABRE | 1.468117371 | 0.023425827 |
| 213 | ALB | 1.465074540 | 0.037002162 |
| 6581 | SLC22A3 | 1.463954569 | 0.016113803 |
| 259 | AMBP | 1.450646810 | 0.017444397 |
| 1842 | ECM2 | 1.448472726 | 0.000147365 |
| 388815 | N/A | 1.446506599 | 0.004335874 |
| 84699 | CREB3L3 | 1.445202911 | 0.024012291 |
| 5106 | PCK2 | 1.443605870 | 0.001587637 |
| 399959 | MIR100HG | 1.436763610 | 0.005262615 |
| 3699 | ITIH3 | 1.433683239 | 0.015408083 |
| 1361 | CPB2 | 1.432397218 | 0.016354400 |
| 50509 | COL5A3 | 1.432109449 | 0.001899974 |
| 27284 | SULT1B1 | 1.430225484 | 0.018618149 |
| 8431 | NR0B2 | 1.429688102 | 0.029235533 |
| 27443 | CECR2 | 1.427703983 | 0.024054631 |
| 29119 | CTNNA3 | 1.426648419 | 0.015006740 |
| 5005 | ORM2 | 1.425526543 | 0.027492056 |
| 6401 | SELE | 1.424234398 | 0.016895563 |
| 6470 | SHMT1 | 1.421016227 | 0.000912600 |
| 4501 | MT1X | 1.420866767 | 0.040183179 |
| 4051 | CYP4F3 | 1.420775249 | 0.015894113 |
| 26238 | LINC01558 | 1.416824790 | 0.005682531 |
| 5004 | ORM1 | 1.415826815 | 0.028064630 |
| 10060 | ABCC9 | 1.415596396 | 0.003366254 |
| 6335 | SCN9A | 1.413961978 | 0.013336958 |
| 7274 | TTPA | 1.413835647 | 0.028802054 |
| 5046 | PCSK6 | 1.409326956 | 0.003610406 |
| 90586 | AOC4P | 1.407813954 | 0.009644706 |
| 338821 | SLCO1B7 | 1.405237364 | 0.019328311 |
| 5950 | RBP4 | 1.402345482 | 0.040406923 |
| 10349 | ABCA10 | 1.400760353 | 0.009108983 |
| 22809 | ATF5 | 1.394730619 | 0.036585322 |
| 10157 | AASS | 1.389967645 | 0.011910273 |
| 79054 | TRPM8 | 1.386587936 | 0.041276147 |
| 56624 | ASAH2 | 1.383674374 | 0.006694146 |
| 79974 | CPED1 | 1.382958699 | 0.009010569 |
| 245 | ALOX12P2 | 1.376601275 | 0.012907206 |
| 84630 | TTBK1 | 1.367764765 | 0.004811105 |
| 6097 | RORC | 1.366878963 | 0.001457820 |
| 8022 | LHX3 | 1.366438412 | 0.047556874 |
| 55107 | ANO1 | 1.360123078 | 0.037338010 |
| 29958 | DMGDH | 1.351341837 | 0.020766218 |
| 3697 | ITIH1 | 1.351313833 | 0.029753442 |
| 4610 | MYCL | 1.350329714 | 0.002795463 |
| 84253 | GARNL3 | 1.350145275 | 0.006583090 |
| 89822 | KCNK17 | 1.348572600 | 0.028163104 |
| 27329 | ANGPTL3 | 1.345721441 | 0.036205928 |
| 388182 | N/A | 1.344102608 | 0.021757967 |
| 84460 | ZMAT1 | 1.340505762 | 0.000361876 |
| 2147 | F2 | 1.335866243 | 0.025530006 |
| 84873 | ADGRG7 | 1.331848934 | 0.027688440 |
| 2277 | VEGFD | 1.325837861 | 0.048006646 |
| 6098 | ROS1 | 1.320258609 | 0.031317343 |
| 2203 | FBP1 | 1.319634995 | 0.037390222 |
| 51181 | DCXR | 1.319467687 | 0.008432687 |
| 83715 | ESPN | 1.313818872 | 0.008096425 |
| 152195 | NUDT16P1 | 1.313575016 | 0.033129674 |
| 199920 | FYB2 | 1.306787520 | 0.023628838 |
| 494470 | RNF165 | 1.306142836 | 0.017249724 |
| 64072 | CDH23 | 1.297260629 | 0.006524927 |
| 10351 | ABCA8 | 1.293599393 | 0.043942392 |
| 57569 | ARHGAP20 | 1.293154723 | 0.013124018 |
| 10144 | FAM13A | 1.291880750 | 0.006341895 |
| 432 | ASGR1 | 1.291283747 | 0.019956348 |
| 26053 | AUTS2 | 1.288010803 | 0.008313303 |
| 285154 | CYP1B1-AS1 | 1.286684001 | 0.007298905 |
| 79632 | FAM184A | 1.280735183 | 0.036457415 |
| 2329 | FMO4 | 1.277863240 | 0.000127037 |
| 5176 | SERPINF1 | 1.275011915 | 0.015883909 |
| 1812 | DRD1 | 1.274528749 | 0.049149818 |
| 57678 | GPAM | 1.271366558 | 0.015807361 |
| 7216 | TRO | 1.270414852 | 0.004374857 |
| 219670 | ENKUR | 1.252328995 | 0.001468013 |
| 349149 | GJC3 | 1.247486863 | 0.007512978 |
| 338323 | NLRP14 | 1.245980146 | 0.007596123 |
| 260293 | CYP4X1 | 1.245825942 | 0.014414288 |
| 3698 | ITIH2 | 1.243055690 | 0.023671309 |
| 246181 | AKR7L | 1.239480534 | 0.008944274 |
| 54923 | LIME1 | 1.239480267 | 0.007733856 |
| 51380 | CSAD | 1.239471506 | 0.000374049 |
| 2819 | GPD1 | 1.231605305 | 0.035276503 |
| 340811 | AKR1C8P | 1.229819596 | 0.037736074 |
| 646023 | ADORA2A-AS1 | 1.229310558 | 0.029797605 |
| 5446 | PON3 | 1.227744138 | 0.028043688 |
| 8824 | CES2 | 1.223262489 | 0.002640713 |
| 7448 | VTN | 1.221142167 | 0.009252379 |
| 84983 | FAM222A-AS1 | 1.220574470 | 0.001968580 |
| 27141 | CIDEB | 1.220094972 | 0.000764360 |
| 377841 | ENTPD8 | 1.220017899 | 0.044330609 |
| 91614 | DEPDC7 | 1.217544996 | 0.025594364 |
| 23650 | TRIM29 | 1.213453057 | 0.039024427 |
| 6334 | SCN8A | 1.213403280 | 0.018486509 |
| 348249 | N/A | 1.209494445 | 0.016954648 |
| 170392 | OIT3 | 1.209213391 | 0.012045028 |
| 55244 | SLC47A1 | 1.209086881 | 0.024281888 |
| 50940 | PDE11A | 1.204782096 | 0.030866602 |
| 100133205 | LINC00240 | 1.203547604 | 0.002058614 |
| 154 | ADRB2 | 1.202106431 | 0.025748268 |
| 8309 | ACOX2 | 1.200327401 | 0.035491968 |
| 8512 | MBL1P | 1.199904359 | 0.019717400 |
| 54873 | PALMD | 1.198587085 | 0.002881576 |
| 51085 | MLXIPL | 1.195836831 | 0.000152905 |
| 761 | CA3 | 1.194667869 | 0.015980601 |
| 10050 | SLC17A4 | 1.191172477 | 0.033406065 |
| 23562 | CLDN14 | 1.187857923 | 0.020602800 |
| 150356 | CHADL | 1.187834368 | 0.034986706 |
| 147 | ADRA1B | 1.186540017 | 0.007194874 |
| 440503 | PLIN5 | 1.185077159 | 0.002948669 |
| 8777 | MPDZ | 1.183643951 | 0.001780448 |
| 1943 | EFNA2 | 1.177989949 | 0.004629949 |
| 130 | ADH6 | 1.175841330 | 0.039125191 |
| 320 | APBA1 | 1.169781308 | 0.023853528 |
| 154661 | RUNDC3B | 1.169078724 | 0.010243899 |
| 5024 | P2RX3 | 1.167963719 | 0.009063695 |
| 153769 | SH3RF2 | 1.167762114 | 0.034071017 |
| 7098 | TLR3 | 1.166028552 | 0.012056776 |
| 3795 | KHK | 1.162429632 | 0.016468594 |
| 2028 | ENPEP | 1.160645083 | 0.001651950 |
| 127254 | ERICH3 | 1.158219563 | 0.017475746 |
| 51156 | SERPINA10 | 1.156496846 | 0.035771487 |
| 6038 | RNASE4 | 1.150719571 | 0.000743368 |
| 79776 | ZFHX4 | 1.150602446 | 0.006145916 |
| 81543 | LRRC3 | 1.149440558 | 0.013476296 |
| 140699 | MROH8 | 1.149300375 | 0.000202225 |
| 57447 | NDRG2 | 1.149264634 | 0.000031873 |
| 285800 | PRR18 | 1.148433442 | 0.02244634 |
| 140828 | LINC00261 | 1.146943493 | 0.012495279 |
| 2780 | GNAT2 | 1.143833603 | 0.004007330 |
| 116844 | LRG1 | 1.143076026 | 0.025846685 |
| 6006 | RHCE | 1.141723830 | 0.042967882 |
| 89886 | SLAMF9 | 1.141445194 | 0.042450137 |
| 286097 | MICU3 | 1.139195435 | 0.011572289 |
| 441476 | STPG3 | 1.137809695 | 0.018937208 |
| 55277 | FGGY | 1.135260604 | 0.038376635 |
| 285954 | INHBA-AS1 | 1.135077363 | 0.011504418 |
| 414152 | C10ORF105 | 1.133765997 | 0.011086218 |
| 57026 | PDXP | 1.133587715 | 7.47276E-05 |
| 18 | ABAT | 1.132396717 | 0.016954011 |
| 54627 | MAP10 | 1.130319302 | 0.023493353 |
| 23500 | DAAM2 | 1.130304113 | 0.011901214 |
| 5651 | TMPRSS15 | 1.122836763 | 0.049493534 |
| 100188954 | N/A | 1.119940555 | 0.030480168 |
| 147525 | N/A | 1.115742152 | 0.005162458 |
| 9104 | RGN | 1.114413160 | 0.037408486 |
| 54084 | TSPEAR | 1.112008850 | 0.043358610 |
| 2155 | F7 | 1.109456476 | 0.008285169 |
| 400752 | N/A | 1.106026795 | 0.000268752 |
| 7704 | ZBTB16 | 1.104977008 | 0.009865461 |
| 653190 | N/A | 1.101097272 | 0.007585401 |
| 1647 | GADD45A | 1.100026962 | 2.00943E-05 |
| 5959 | RDH5 | 1.098574216 | 0.007042391 |
| 401288 | LINC00242 | 1.098269741 | 0.001299677 |
| 100129387 | N/A | 1.096172325 | 0.000282967 |
| 2052 | EPHX1 | 1.093699058 | 0.015414286 |
| 57134 | MAN1C1 | 1.088347318 | 0.031088089 |
| 89927 | C16ORF45 | 1.085595990 | 0.005447660 |
| 54757 | FAM20A | 1.082322076 | 0.016216782 |
| 254427 | PROSER2 | 1.081743733 | 0.007964361 |
| 7425 | VGF | 1.081531810 | 0.008800112 |
| 6652 | SORD | 1.080382330 | 0.020779186 |
| 342931 | RFPL4A | 1.080173565 | 0.040723161 |
| 377677 | CA13 | 1.079310178 | 0.009919664 |
| 115817 | DHRS1 | 1.074820067 | 0.001006992 |
| 122622 | ADSSL1 | 1.074129809 | 0.009184543 |
| 10402 | ST3GAL6 | 1.072964688 | 0.007123780 |
| 6095 | RORA | 1.071228748 | 7.62058E-05 |
| 4843 | NOS2 | 1.066715913 | 0.041703698 |
| 3638 | INSIG1 | 1.064815107 | 0.038862707 |
| 32 | ACACB | 1.059698185 | 0.001686509 |
| 3075 | CFH | 1.055741774 | 0.030385611 |
| 1593 | CYP27A1 | 1.052799928 | 0.029272041 |
| 84217 | ZMYND12 | 1.051651416 | 0.036009590 |
| 285386 | TPRG1 | 1.048592099 | 0.031583257 |
| 79660 | PPP1R3B | 1.048426658 | 0.004569388 |
| 391059 | FRRS1 | 1.047308419 | 0.035947478 |
| 978 | CDA | 1.042511538 | 0.047707531 |
| 718 | C3 | 1.042156504 | 0.017853286 |
| 8542 | APOL1 | 1.039415681 | 0.002420486 |
| 84952 | CGNL1 | 1.038563740 | 0.020445663 |
| 79614 | N/A | 1.037537142 | 0.041356081 |
| 79814 | AGMAT | 1.036960620 | 0.048040584 |
| 131 | ADH7 | 1.035696434 | 0.038098856 |
| 64284 | RAB17 | 1.034620697 | 0.023942950 |
| 10826 | FAXDC2 | 1.034324678 | 0.002391724 |
| 23600 | AMACR | 1.033956168 | 0.032624265 |
| 149465 | CFAP57 | 1.033364636 | 0.008166289 |
| 279 | AMY2A | 1.033321944 | 0.020321949 |
| 1757 | SARDH | 1.032628652 | 0.015510262 |
| 3226 | HOXC10 | 1.031904017 | 0.037868401 |
| 5380 | N/A | 1.028439705 | 0.000293284 |
| 4522 | MTHFD1 | 1.023805714 | 0.002358802 |
| 3577 | CXCR1 | 1.023780695 | 0.025209753 |
| 220004 | PPP1R32 | 1.022166053 | 0.008785035 |
| 285704 | RGMB | 1.021856316 | 0.004444710 |
| 399671 | HEATR4 | 1.018090387 | 0.019794184 |
| 116 | ADCYAP1 | 1.016673106 | 0.046225573 |
| 116255 | MOGAT1 | 1.014613791 | 0.009106562 |
| 6567 | SLC16A2 | 1.013941147 | 0.005829829 |
| 6342 | SCP2 | 1.012230142 | 0.000588239 |
| 28959 | TMEM176B | 1.010920124 | 0.019629853 |
| 284723 | SLC25A34 | 1.009042952 | 0.027160020 |
| 368 | ABCC6 | 1.005286833 | 0.008909763 |
| 5919 | RARRES2 | 1.004067478 | 0.011290875 |
| 79729 | SH3D21 | -1.001123830 | 0.004751775 |
| 991 | CDC20 | -1.002760237 | 0.025530959 |
| 57468 | SLC12A5 | -1.004562500 | 0.008673047 |
| 84688 | C9ORF24 | -1.005609921 | 0.009267502 |
| 770 | CA11 | -1.006909647 | 0.026512726 |
| 9454 | HOMER3 | -1.007842144 | 0.010329995 |
| 80763 | SPX | -1.009970370 | 0.047386534 |
| 54704 | PDP1 | -1.010436285 | 0.007250862 |
| 400629 | TEX19 | -1.011514889 | 0.045583694 |
| 644 | BLVRA | -1.014072197 | 0.004980499 |
| 57655 | GRAMD1A | -1.020483391 | 0.000855917 |
| 9956 | HS3ST2 | -1.021026784 | 0.025808302 |
| 1237 | CCR8 | -1.025273843 | 0.007499397 |
| 636 | BICD1 | -1.025858787 | 0.014533123 |
| 287 | ANK2 | -1.028256091 | 0.029530984 |
| 4130 | MAP1A | -1.028837764 | 0.044791204 |
| 4619 | MYH1 | -1.032697225 | 0.045731960 |
| 4828 | NMB | -1.033638898 | 0.009623243 |
| 4118 | MAL | -1.034000160 | 0.030564307 |
| 9805 | SCRN1 | -1.034811618 | 0.034065097 |
| 84662 | GLIS2 | -1.037043451 | 0.022253202 |
| 85479 | DNAJC5B | -1.040909474 | 0.047583649 |
| 9966 | TNFSF15 | -1.043460505 | 0.032033579 |
| 6887 | TAL2 | -1.043485639 | 0.020746944 |
| 777 | CACNA1E | -1.046013803 | 0.046354211 |
| 941 | CD80 | -1.047868233 | 0.026317087 |
| 9170 | LPAR2 | -1.048460085 | 0.028810306 |
| 343263 | MYBPHL | -1.048878260 | 0.026680468 |
| 84900 | RNFT2 | -1.049507583 | 0.011840666 |
| 55138 | FAM90A1 | -1.052241448 | 0.016488239 |
| 27306 | HPGDS | -1.053381493 | 0.018726789 |
| 57562 | CEP126 | -1.058066118 | 0.032615304 |
| 4692 | NDN | -1.059132713 | 0.031835214 |
| 26999 | CYFIP2 | -1.059893426 | 0.033356281 |
| 79801 | SHCBP1 | -1.063529208 | 0.030686572 |
| 9654 | TTLL4 | -1.064128284 | 0.008864083 |
| 26996 | GPR160 | -1.064407350 | 0.005775609 |
| 401884 | N/A | -1.064814446 | 0.022253886 |
| 79888 | LPCAT1 | -1.077109474 | 0.002447006 |
| 92558 | BICDL1 | -1.084870164 | 0.030350761 |
| 11322 | TMC6 | -1.085514659 | 0.005820871 |
| 91373 | UAP1L1 | -1.085598696 | 0.025285397 |
| 26150 | RIBC2 | -1.093757714 | 0.040076184 |
| 4050 | LTB | -1.094388431 | 0.039634298 |
| 342184 | FMN1 | -1.094884045 | 0.012581329 |
| 6875 | TAF4B | -1.102864521 | 0.015079077 |
| 50615 | IL21R | -1.103329043 | 0.043109335 |
| 84842 | HPDL | -1.103887810 | 0.039626806 |
| 7040 | TGFB1 | -1.106259810 | 0.006099409 |
| 1235 | CCR6 | -1.107584342 | 0.024493721 |
| 84941 | HSH2D | -1.111837698 | 0.029570408 |
| 9022 | CLIC3 | -1.118537954 | 0.045822506 |
| 23302 | WSCD1 | -1.119734191 | 0.028783814 |
| 10518 | CIB2 | -1.122919185 | 0.031744582 |
| 84935 | MEDAG | -1.124468832 | 0.039571866 |
| 23363 | OBSL1 | -1.127672929 | 0.032755606 |
| 84959 | UBASH3B | -1.128549082 | 0.004436883 |
| 9143 | SYNGR3 | -1.132121288 | 0.046934128 |
| 23612 | PHLDA3 | -1.132435560 | 0.033744693 |
| 8601 | RGS20 | -1.134452308 | 0.009790580 |
| 6785 | ELOVL4 | -1.136825340 | 0.007128961 |
| 26230 | TIAM2 | -1.136977286 | 0.018871806 |
| 1535 | CYBA | -1.139286416 | 0.014296784 |
| 27090 | ST6GALNAC4 | -1.146072950 | 0.000500887 |
| 11077 | HSF2BP | -1.149298465 | 0.010983236 |
| 162979 | ZNF296 | -1.151637890 | 0.012408152 |
| 23426 | GRIP1 | -1.164663296 | 0.041407039 |
| 388242 | N/A | -1.166220886 | 0.008689477 |
| 27202 | C5AR2 | -1.166482110 | 0.036878321 |
| 776 | CACNA1D | -1.169345714 | 0.015516530 |
| 9545 | RAB3D | -1.172134765 | 0.026886525 |
| 256236 | NAPSB | -1.175276449 | 0.031322095 |
| 55314 | TMEM144 | -1.179217810 | 0.038003221 |
| 9518 | GDF15 | -1.186086909 | 0.014998885 |
| 113157 | RPLP0P2 | -1.186220842 | 0.008202717 |
| 646851 | FAM227A | -1.187099076 | 0.015739533 |
| 414236 | C10ORF55 | -1.195957796 | 0.001786196 |
| 92521 | SPECC1 | -1.197404700 | 0.021947882 |
| 2980 | GUCA2A | -1.197585716 | 0.044589670 |
| 55789 | DEPDC1B | -1.199399506 | 0.028548115 |
| 55282 | LRRC36 | -1.200214323 | 0.013313551 |
| 924 | CD7 | -1.201465552 | 0.014086465 |
| 400745 | SH2D5 | -1.208124794 | 0.011782893 |
| 83850 | ESYT3 | -1.208825823 | 0.029734107 |
| 23066 | CAND2 | -1.212122476 | 0.019659493 |
| 10360 | NPM3 | -1.220103263 | 0.000654042 |
| 23529 | CLCF1 | -1.221402840 | 0.009672020 |
| 57801 | HES4 | -1.222554645 | 0.032596815 |
| 84519 | ACRBP | -1.223657882 | 0.008019256 |
| 4192 | MDK | -1.224639048 | 0.025373945 |
| 576 | ADGRB2 | -1.229399285 | 0.047938818 |
| 1298 | COL9A2 | -1.229597063 | 0.045726575 |
| 3684 | ITGAM | -1.238551841 | 0.006721281 |
| 9856 | KIAA0319 | -1.243688085 | 0.032705162 |
| 145200 | LINC00239 | -1.248696381 | 0.023904054 |
| 747 | DAGLA | -1.254390877 | 0.012672595 |
| 25827 | FBXL2 | -1.256705303 | 0.020227488 |
| 64220 | STRA6 | -1.259405842 | 0.027283065 |
| 55529 | PIP4P2 | -1.262381595 | 0.000696795 |
| 6510 | SLC1A5 | -1.266859375 | 0.005770650 |
| 407977 | TNFSF12-TNFSF13 | -1.268614280 | 0.030778030 |
| 23025 | UNC13A | -1.268929805 | 0.037057878 |
| 1493 | CTLA4 | -1.271554687 | 0.029133810 |
| 113828 | FAM83F | -1.272894852 | 0.048424928 |
| 54503 | ZDHHC13 | -1.273068318 | 0.002071660 |
| 10863 | ADAM28 | -1.273624748 | 0.024199290 |
| 7220 | TRPC1 | -1.280881195 | 0.012472986 |
| 3691 | ITGB4 | -1.281794737 | 0.010026231 |
| 23682 | RAB38 | -1.283349609 | 0.014900612 |
| 7941 | PLA2G7 | -1.283920840 | 0.004060545 |
| 7301 | TYRO3 | -1.288235317 | 0.019781783 |
| 1400 | CRMP1 | -1.292107411 | 0.008018379 |
| 57722 | IGDCC4 | -1.292551910 | 0.035936175 |
| 80003 | PCNX2 | -1.299012220 | 0.021772175 |
| 3159 | HMGA1 | -1.302539570 | 9.75976E-05 |
| 132884 | EVC2 | -1.311132937 | 0.040614555 |
| 57643 | ZSWIM5 | -1.311689149 | 0.037981260 |
| 9456 | HOMER1 | -1.313607369 | 0.026141901 |
| 9200 | HACD1 | -1.315112366 | 0.044482718 |
| 497189 | TIFAB | -1.315389465 | 0.006037284 |
| 6277 | S100A6 | -1.316791843 | 0.036219682 |
| 2539 | G6PD | -1.317048445 | 0.001703053 |
| 388931 | MFSD2B | -1.317983859 | 0.000771080 |
| 89832 | CHRFAM7A | -1.321601004 | 0.004278256 |
| 26011 | TENM4 | -1.325369943 | 0.029844517 |
| 161582 | DNAAF4 | -1.330540034 | 0.005387249 |
| 114801 | TMEM200A | -1.333459974 | 0.007894232 |
| 80736 | SLC44A4 | -1.338530263 | 0.040276028 |
| 29108 | PYCARD | -1.346765780 | 0.009911439 |
| 55561 | CDC42BPG | -1.347265387 | 0.045563509 |
| 284340 | CXCL17 | -1.348075016 | 0.008394606 |
| 57692 | MAGEE1 | -1.349268940 | 0.001380695 |
| 80023 | NRSN2 | -1.349374670 | 0.005209377 |
| 55423 | SIRPG | -1.351306906 | 0.025639179 |
| 11074 | TRIM31 | -1.355253758 | 0.048876061 |
| 57628 | DPP10 | -1.355781160 | 0.041720420 |
| 348378 | FAM159A | -1.367707070 | 0.019564590 |
| 55227 | LRRC1 | -1.367938683 | 0.023575252 |
| 1117 | CHI3L2 | -1.369250662 | 0.049870026 |
| 6689 | SPIB | -1.380488312 | 0.027648136 |
| 4355 | MPP2 | -1.381624013 | 0.008745042 |
| 375616 | KCP | -1.383412950 | 0.022425565 |
| 4148 | MATN3 | -1.388748986 | 0.043829176 |
| 6273 | S100A2 | -1.388918665 | 0.019282009 |
| 933 | CD22 | -1.390885285 | 0.033099725 |
| 54509 | RHOF | -1.392194491 | 0.034767123 |
| 114804 | RNF157 | -1.393246320 | 0.013949287 |
| 84264 | HAGHL | -1.393277939 | 0.046310798 |
| 5450 | POU2AF1 | -1.400989560 | 0.039910143 |
| 79734 | KCTD17 | -1.404570401 | 0.006572265 |
| 8877 | SPHK1 | -1.404672875 | 0.024513890 |
| 1608 | DGKG | -1.404689961 | 0.023411239 |
| 90853 | SPOCD1 | -1.407767405 | 0.005753487 |
| 114787 | GPRIN1 | -1.410402506 | 0.007884511 |
| 54478 | PIMREG | -1.421664708 | 0.005802751 |
| 6349 | CCL3L3 | -1.425353660 | 0.006302968 |
| 9609 | RAB36 | -1.430160057 | 0.030897510 |
| 5973 | RENBP | -1.439057913 | 0.009121925 |
| 2151 | F2RL2 | -1.448988807 | 0.026650913 |
| 10626 | TRIM16 | -1.452216216 | 0.012670900 |
| 51127 | TRIM17 | -1.456132775 | 0.016920904 |
| 148113 | CILP2 | -1.463695141 | 0.037710396 |
| 131405 | TRIM71 | -1.465014572 | 0.015819164 |
| 84033 | OBSCN | -1.474618786 | 0.012984586 |
| 83871 | RAB34 | -1.474939160 | 0.002970477 |
| 55356 | SLC22A15 | -1.475077726 | 0.025600542 |
| 5270 | SERPINE2 | -1.484552495 | 0.024661293 |
| 5754 | PTK7 | -1.485693048 | 0.008529629 |
| 167838 | TXLNB | -1.489605993 | 0.006420802 |
| 255231 | MCOLN2 | -1.496152338 | 0.010100331 |
| 84221 | SPATC1L | -1.496327558 | 0.017820701 |
| 93082 | NEURL3 | -1.497581535 | 0.040512603 |
| 79805 | VASH2 | -1.506449199 | 0.015376490 |
| 23057 | NMNAT2 | -1.509176077 | 0.010704926 |
| 6338 | SCNN1B | -1.522652350 | 0.010654044 |
| 222962 | SLC29A4 | -1.525102697 | 0.048114919 |
| 6375 | XCL1 | -1.530709383 | 0.001339958 |
| 92086 | GGTLC1 | -1.540560738 | 0.011919372 |
| 150 | ADRA2A | -1.542528983 | 0.016362646 |
| 2191 | FAP | -1.547925234 | 0.015009840 |
| 22996 | TTC39A | -1.552459263 | 0.038058394 |
| 8038 | ADAM12 | -1.553417812 | 0.022793618 |
| 780 | DDR1 | -1.555946838 | 0.012187931 |
| 81793 | TLR10 | -1.557892672 | 0.007600218 |
| 6541 | SLC7A1 | -1.569101562 | 0.003991509 |
| 147645 | VSIG10L | -1.569650730 | 0.002028702 |
| 26027 | ACOT11 | -1.572772812 | 0.007549676 |
| 619373 | MBOAT4 | -1.574093059 | 0.008649112 |
| 8745 | ADAM23 | -1.581051475 | 0.044191100 |
| 90993 | CREB3L1 | -1.594216574 | 0.024166104 |
| 8712 | PAGE1 | -1.602764329 | 0.040192107 |
| 83879 | CDCA7 | -1.626855103 | 0.018210848 |
| 51207 | DUSP13 | -1.668968611 | 0.006191255 |
| 1410 | CRYAB | -1.672400633 | 0.022559920 |
| 3957 | LGALS2 | -1.682658945 | 0.005973089 |
| 23213 | SULF1 | -1.683020713 | 0.008916165 |
| 169044 | COL22A1 | -1.687711872 | 0.037154477 |
| 64208 | POPDC3 | -1.697012069 | 0.020110012 |
| 26279 | PLA2G2D | -1.707557127 | 0.026349379 |
| 57476 | GRAMD1B | -1.717122865 | 0.044382573 |
| 10631 | POSTN | -1.720776750 | 0.042113235 |
| 55630 | SLC39A4 | -1.721344593 | 0.023717347 |
| 333 | APLP1 | -1.724916584 | 0.029659163 |
| 135295 | SRSF12 | -1.755683062 | 0.000750670 |
| 4320 | MMP11 | -1.762124576 | 0.001023686 |
| 4318 | MMP9 | -1.787391604 | 0.008917117 |
| 112609 | MRAP2 | -1.805823671 | 0.026185068 |
| 5831 | PYCR1 | -1.808100754 | 0.028409566 |
| 26256 | CABYR | -1.810042328 | 0.040877628 |
| 80201 | HKDC1 | -1.830744644 | 0.028509176 |
| 50804 | MYEF2 | -1.833725552 | 0.015466750 |
| 6819 | SULT1C2 | -1.836897601 | 0.045437191 |
| 54997 | TESC | -1.856617454 | 0.008847670 |
| 6297 | SALL2 | -1.860438165 | 0.001466065 |
| 79695 | GALNT12 | -1.865928632 | 0.006563507 |
| 2679 | N/A | -1.870310376 | 0.001557157 |
| 58473 | PLEKHB1 | -1.872564506 | 0.006206063 |
| 125931 | CEACAM20 | -1.878921087 | 0.013203085 |
| 51454 | GULP1 | -1.885698371 | 0.035325981 |
| 4321 | MMP12 | -1.885756332 | 0.011100794 |
| 771 | CA12 | -1.893527267 | 0.032172920 |
| 144501 | KRT80 | -1.894940548 | 0.040942869 |
| 1014 | CDH16 | -1.898936980 | 0.019275299 |
| 94234 | FOXQ1 | -1.906900635 | 0.015768701 |
| 9536 | PTGES | -1.909019525 | 0.036247638 |
| 284217 | LAMA1 | -1.910128931 | 0.017895414 |
| 91227 | GGTLC2 | -1.910946717 | 0.004041093 |
| 80144 | FRAS1 | -1.911712138 | 0.025669935 |
| 2302 | FOXJ1 | -1.916580174 | 0.020127655 |
| 26762 | HAVCR1 | -1.948499845 | 0.003980754 |
| 89780 | WNT3A | -1.950715160 | 0.019441743 |
| 8153 | RND2 | -1.960796744 | 0.017304413 |
| 5225 | PGC | -1.995596241 | 0.043822363 |
| 2678 | GGT1 | -1.998697563 | 0.001582440 |
| 253982 | ASPHD1 | -2.020689877 | 0.028169749 |
| 51237 | MZB1 | -2.031198370 | 0.019798276 |
| 23657 | SLC7A11 | -2.037554309 | 0.004994662 |
| 115584 | SLC5A11 | -2.064372136 | 0.021383189 |
| 312 | ANXA13 | -2.066443098 | 0.038404654 |
| 84302 | TMEM246 | -2.102125265 | 0.000683001 |
| 25817 | FAM19A5 | -2.111997026 | 0.027111090 |
| 27299 | ADAMDEC1 | -2.112209899 | 0.000884125 |
| 5603 | MAPK13 | -2.125895493 | 0.001565814 |
| 2318 | FLNC | -2.166737816 | 0.012697588 |
| 26470 | SEZ6L2 | -2.182053748 | 0.020941236 |
| 4440 | MSI1 | -2.197551129 | 0.012551362 |
| 126353 | MISP | -2.240727761 | 0.017424813 |
| 23532 | PRAME | -2.243818832 | 0.025079322 |
| 9245 | GCNT3 | -2.295442487 | 0.008008837 |
| 54825 | CDHR2 | -2.357085765 | 0.043317308 |
| 220963 | SLC16A9 | -2.365148310 | 0.024378399 |
| 2984 | GUCY2C | -2.504026110 | 0.009164089 |
| 3352 | HTR1D | -2.508409024 | 0.003310099 |
| 65268 | WNK2 | -2.656948548 | 0.014960929 |
| 22943 | DKK1 | -2.666057251 | 0.004906304 |
| 4072 | EPCAM | -2.682699836 | 0.036029350 |
| 1515 | CTSV | -2.699439321 | 0.000143908 |
| 10642 | IGF2BP1 | -2.713113555 | 0.006518789 |
| 1728 | NQO1 | -2.736614322 | 0.004408202 |
| 6696 | SPP1 | -2.740393786 | 0.016666969 |
