# Supplementary material for: Dual prognostic role of 2-oxoglutarate-dependent oxygenases in ten cancer types: implications for cell cycle regulation and cell adhesion maintenance
Source: Cancer Commun (Lond). 2019 Apr 29;39:23. doi: 10.1186/s40880-019-0369-5 (PMC6489267; doi:10.1186/s40880-019-0369-5)
Supplement: Supplementary file 9 — Additional file 9. Significantly enriched biological pathways of differentially expressed genes. [file 40880_2019_369_MOESM9_ESM.docx]

| **Additional File 9. Significantly enriched biological pathways of differentially expressed genes.** | | | |
| --- | --- | --- | --- |
|  |  |  |  |
| **Category** | **Ontology** | ***P* Value** | **Fold Enrichment** |
| GO_biological_processes | GO:0006805~xenobiotic metabolic process | 3.46E-17 | 8.87 |
| GO_biological_processes | GO:0055114~oxidation-reduction process | 5.66E-15 | 2.97 |
| KEGG_pathway | hsa01100:Metabolic pathways | 1.26E-13 | 1.96 |
| KEGG_pathway | hsa04610:Complement and coagulation cascades | 1.18E-11 | 6.68 |
| GO_biological_processes | GO:0006699~bile acid biosynthetic process | 4.77E-11 | 15.21 |
| KEGG_pathway | hsa00830:Retinol metabolism | 2.51E-09 | 6.07 |
| GO_biological_processes | GO:0010951~negative regulation of endopeptidase activity | 4.15E-09 | 4.84 |
| GO_biological_processes | GO:0019373~epoxygenase P450 pathway | 5.00E-09 | 14.78 |
| KEGG_pathway | hsa05204:Chemical carcinogenesis | 1.15E-08 | 5.21 |
| KEGG_pathway | hsa00120:Primary bile acid biosynthesis | 1.30E-08 | 12.90 |
| KEGG_pathway | hsa00980:Metabolism of xenobiotics by cytochrome P450 | 2.12E-08 | 5.34 |
| GO_biological_processes | GO:0008202~steroid metabolic process | 3.70E-08 | 8.05 |
| KEGG_pathway | hsa00982:Drug metabolism - cytochrome P450 | 3.87E-08 | 5.48 |
| KEGG_pathway | hsa04976:Bile secretion | 4.83E-08 | 5.40 |
| GO_biological_processes | GO:0042738~exogenous drug catabolic process | 6.80E-08 | 17.74 |
| GO_biological_processes | GO:0006869~lipid transport | 1.18E-07 | 5.60 |
| GO_biological_processes | GO:0070328~triglyceride homeostasis | 2.45E-07 | 10.24 |
| GO_biological_processes | GO:0017144~drug metabolic process | 3.56E-07 | 9.86 |
| GO_biological_processes | GO:0055085~transmembrane transport | 5.19E-07 | 3.05 |
| GO_biological_processes | GO:0015721~bile acid and bile salt transport | 4.43E-06 | 8.87 |
| KEGG_pathway | hsa03320:PPAR signaling pathway | 7.57E-06 | 4.58 |
| GO_biological_processes | GO:0001523~retinoid metabolic process | 1.44E-05 | 5.24 |
| GO_biological_processes | GO:0006631~fatty acid metabolic process | 1.96E-05 | 5.63 |
| KEGG_pathway | hsa00071:Fatty acid degradation | 2.03E-05 | 5.48 |
| GO_biological_processes | GO:0002576~platelet degranulation | 2.99E-05 | 3.88 |
| GO_biological_processes | GO:0019432~triglyceride biosynthetic process | 3.57E-05 | 8.19 |
| KEGG_pathway | hsa00010:Glycolysis / Gluconeogenesis | 3.96E-05 | 4.26 |
| GO_biological_processes | GO:0006069~ethanol oxidation | 4.65E-05 | 13.31 |
| KEGG_pathway | hsa00260:Glycine, serine and threonine metabolism | 4.67E-05 | 5.62 |
| GO_biological_processes | GO:0042632~cholesterol homeostasis | 1.25E-04 | 4.57 |
| KEGG_pathway | hsa00350:Tyrosine metabolism | 1.33E-04 | 5.64 |
| GO_biological_processes | GO:0051919~positive regulation of fibrinolysis | 2.04E-04 | 26.61 |
| GO_biological_processes | GO:0097267~omega-hydroxylase P450 pathway | 2.13E-04 | 14.78 |
| GO_biological_processes | GO:0009636~response to toxic substance | 3.26E-04 | 3.76 |
| KEGG_pathway | hsa04146:Peroxisome | 3.33E-04 | 3.44 |
| GO_biological_processes | GO:0045723~positive regulation of fatty acid biosynthetic process | 3.44E-04 | 13.31 |
| GO_biological_processes | GO:0043691~reverse cholesterol transport | 4.17E-04 | 8.87 |
| GO_biological_processes | GO:0007597~blood coagulation, intrinsic pathway | 4.17E-04 | 8.87 |
| KEGG_pathway | hsa00590:Arachidonic acid metabolism | 4.26E-04 | 3.89 |
| GO_biological_processes | GO:0042157~lipoprotein metabolic process | 4.63E-04 | 5.60 |
| GO_biological_processes | GO:0051180~vitamin transport | 4.97E-04 | 21.29 |
| GO_biological_processes | GO:0006071~glycerol metabolic process | 5.25E-04 | 12.10 |
| GO_biological_processes | GO:0006953~acute-phase response | 5.46E-04 | 5.46 |
| KEGG_pathway | hsa00500:Starch and sucrose metabolism | 5.75E-04 | 5.32 |
| KEGG_pathway | hsa01130:Biosynthesis of antibiotics | 5.96E-04 | 2.28 |
| GO_biological_processes | GO:0006810~transport | 6.94E-04 | 2.06 |
| KEGG_pathway | hsa02010:ABC transporters | 7.03E-04 | 4.49 |
| GO_biological_processes | GO:0030449~regulation of complement activation | 7.48E-04 | 6.21 |
| GO_biological_processes | GO:0042730~fibrinolysis | 9.03E-04 | 7.60 |
| GO_biological_processes | GO:0016098~monoterpenoid metabolic process | 9.66E-04 | 17.74 |
| GO_biological_processes | GO:0042737~drug catabolic process | 9.66E-04 | 17.74 |
| GO_biological_processes | GO:0006957~complement activation, alternative pathway | 0.001070284 | 10.24 |
| GO_biological_processes | GO:0006637~acyl-CoA metabolic process | 0.001132542 | 7.26 |
| GO_biological_processes | GO:0043252~sodium-independent organic anion transport | 0.001402936 | 6.94 |
| GO_biological_processes | GO:0051289~protein homotetramerization | 0.001717738 | 3.99 |
| GO_biological_processes | GO:0015695~organic cation transport | 0.001924609 | 8.87 |
| GO_biological_processes | GO:0008209~androgen metabolic process | 0.00249067 | 8.32 |
| GO_biological_processes | GO:0010898~positive regulation of triglyceride catabolic process | 0.00255538 | 13.31 |
| GO_biological_processes | GO:0006633~fatty acid biosynthetic process | 0.003122356 | 4.09 |
| GO_biological_processes | GO:0055091~phospholipid homeostasis | 0.003726728 | 11.83 |
| GO_biological_processes | GO:0031638~zymogen activation | 0.003726728 | 11.83 |
| GO_biological_processes | GO:0007596~blood coagulation | 0.004023522 | 2.31 |
| GO_biological_processes | GO:0050746~regulation of lipoprotein metabolic process | 0.004111183 | 26.61 |
| GO_biological_processes | GO:0030573~bile acid catabolic process | 0.004111183 | 26.61 |
| KEGG_pathway | hsa00983:Drug metabolism - other enzymes | 0.004345618 | 3.82 |
| GO_biological_processes | GO:0008152~metabolic process | 0.004412615 | 2.38 |
| GO_biological_processes | GO:0042593~glucose homeostasis | 0.004607137 | 2.90 |
| GO_biological_processes | GO:0042572~retinol metabolic process | 0.004786067 | 5.32 |
| GO_biological_processes | GO:0006730~one-carbon metabolic process | 0.004786067 | 5.32 |
| GO_biological_processes | GO:0006855~drug transmembrane transport | 0.004851097 | 7.00 |
| KEGG_pathway | hsa01200:Carbon metabolism | 0.005030137 | 2.52 |
| KEGG_pathway | hsa04922:Glucagon signaling pathway | 0.005057234 | 2.66 |
| GO_biological_processes | GO:0051156~glucose 6-phosphate metabolic process | 0.005176482 | 10.64 |
| GO_biological_processes | GO:0046470~phosphatidylcholine metabolic process | 0.005176482 | 10.64 |
| GO_biological_processes | GO:0006000~fructose metabolic process | 0.005176482 | 10.64 |
| GO_biological_processes | GO:0031667~response to nutrient levels | 0.005533575 | 5.15 |
| GO_biological_processes | GO:0006094~gluconeogenesis | 0.005710899 | 4.23 |
| KEGG_pathway | hsa00910:Nitrogen metabolism | 0.006220473 | 6.45 |
| GO_biological_processes | GO:0007584~response to nutrient | 0.006410732 | 3.24 |
| GO_biological_processes | GO:0017187~peptidyl-glutamic acid carboxylation | 0.00692097 | 9.68 |
| GO_biological_processes | GO:0010867~positive regulation of triglyceride biosynthetic process | 0.00692097 | 9.68 |
| GO_biological_processes | GO:0032094~response to food | 0.007059136 | 6.34 |
| GO_biological_processes | GO:0001676~long-chain fatty acid metabolic process | 0.007059136 | 6.34 |
| GO_biological_processes | GO:0006651~diacylglycerol biosynthetic process | 0.008017984 | 19.96 |
| GO_biological_processes | GO:0019448~L-cysteine catabolic process | 0.008017984 | 19.96 |
| GO_biological_processes | GO:0036101~leukotriene B4 catabolic process | 0.008017984 | 19.96 |
| GO_biological_processes | GO:0010897~negative regulation of triglyceride catabolic process | 0.008017984 | 19.96 |
| GO_biological_processes | GO:0015711~organic anion transport | 0.008973455 | 8.87 |
| GO_biological_processes | GO:0030212~hyaluronan metabolic process | 0.008973455 | 8.87 |
| GO_biological_processes | GO:0070989~oxidative demethylation | 0.008973455 | 8.87 |
| GO_biological_processes | GO:0007586~digestion | 0.009074707 | 3.38 |
| GO_biological_processes | GO:0042594~response to starvation | 0.009351338 | 4.56 |
| KEGG_pathway | hsa05150:Staphylococcus aureus infection | 0.010557477 | 3.25 |
| GO_biological_processes | GO:0051384~response to glucocorticoid | 0.010712715 | 3.28 |
| GO_biological_processes | GO:0042493~response to drug | 0.01097762 | 1.84 |
| KEGG_pathway | hsa04973:Carbohydrate digestion and absorption | 0.011053965 | 3.66 |
| GO_biological_processes | GO:0019915~lipid storage | 0.011469433 | 5.54 |
| GO_biological_processes | GO:0007188~adenylate cyclase-modulating G-protein coupled receptor signaling pathway | 0.011811436 | 4.32 |
| GO_biological_processes | GO:0007204~positive regulation of cytosolic calcium ion concentration | 0.01214725 | 2.38 |
| GO_biological_processes | GO:0015812~gamma-aminobutyric acid transport | 0.013032193 | 15.97 |
| GO_biological_processes | GO:0019439~aromatic compound catabolic process | 0.013032193 | 15.97 |
| GO_biological_processes | GO:0002682~regulation of immune system process | 0.013032193 | 15.97 |
| GO_biological_processes | GO:0033344~cholesterol efflux | 0.013257496 | 5.32 |
| KEGG_pathway | hsa00051:Fructose and mannose metabolism | 0.013760322 | 4.11 |
| GO_biological_processes | GO:0006508~proteolysis | 0.013913326 | 1.60 |
| GO_biological_processes | GO:0033700~phospholipid efflux | 0.014041733 | 7.60 |
| GO_biological_processes | GO:0042573~retinoic acid metabolic process | 0.014041733 | 7.60 |
| GO_biological_processes | GO:0007155~cell adhesion | 0.014451138 | 1.62 |
| GO_biological_processes | GO:0046487~glyoxylate metabolic process | 0.015213524 | 5.12 |
| GO_biological_processes | GO:0006629~lipid metabolic process | 0.015330745 | 2.20 |
| GO_biological_processes | GO:0006691~leukotriene metabolic process | 0.017071014 | 7.10 |
| GO_biological_processes | GO:0034375~high-density lipoprotein particle remodeling | 0.017071014 | 7.10 |
| GO_biological_processes | GO:0010575~positive regulation of vascular endothelial growth factor production | 0.017342128 | 4.93 |
| GO_biological_processes | GO:0006749~glutathione metabolic process | 0.018035879 | 3.33 |
| KEGG_pathway | hsa00232:Caffeine metabolism | 0.01878889 | 13.16 |
| GO_biological_processes | GO:0042426~choline catabolic process | 0.019065477 | 13.31 |
| GO_biological_processes | GO:0009804~coumarin metabolic process | 0.019065477 | 13.31 |
| GO_biological_processes | GO:0006739~NADP metabolic process | 0.019065477 | 13.31 |
| GO_biological_processes | GO:0046483~heterocycle metabolic process | 0.019065477 | 13.31 |
| GO_biological_processes | GO:0008206~bile acid metabolic process | 0.020435709 | 6.65 |
| GO_biological_processes | GO:0006898~receptor-mediated endocytosis | 0.023314405 | 2.00 |
| KEGG_pathway | hsa04931:Insulin resistance | 0.024468004 | 2.23 |
| GO_biological_processes | GO:0097264~self proteolysis | 0.026034557 | 11.41 |
| GO_biological_processes | GO:0010890~positive regulation of sequestering of triglyceride | 0.026034557 | 11.41 |
| GO_biological_processes | GO:0042908~xenobiotic transport | 0.026034557 | 11.41 |
| GO_biological_processes | GO:0015872~dopamine transport | 0.026034557 | 11.41 |
| GO_biological_processes | GO:0001867~complement activation, lectin pathway | 0.026034557 | 11.41 |
| GO_biological_processes | GO:0044539~long-chain fatty acid import | 0.026034557 | 11.41 |
| KEGG_pathway | hsa00270:Cysteine and methionine metabolism | 0.02751594 | 3.46 |
| GO_biological_processes | GO:0060384~innervation | 0.028175749 | 5.91 |
| GO_biological_processes | GO:0045766~positive regulation of angiogenesis | 0.029031787 | 2.31 |
| GO_biological_processes | GO:0050727~regulation of inflammatory response | 0.030367785 | 2.96 |
| KEGG_pathway | hsa04975:Fat digestion and absorption | 0.030429688 | 3.37 |
| KEGG_pathway | hsa00650:Butanoate metabolism | 0.032142023 | 4.06 |
| GO_biological_processes | GO:0006814~sodium ion transport | 0.032219408 | 2.63 |
| GO_biological_processes | GO:0048260~positive regulation of receptor-mediated endocytosis | 0.032549122 | 5.60 |
| GO_biological_processes | GO:0030198~extracellular matrix organization | 0.033745694 | 1.90 |
| GO_biological_processes | GO:0090181~regulation of cholesterol metabolic process | 0.033860949 | 9.98 |
| KEGG_pathway | hsa00460:Cyanoamino acid metabolism | 0.037147063 | 9.40 |
| GO_biological_processes | GO:0019370~leukotriene biosynthetic process | 0.037254292 | 5.32 |
| GO_biological_processes | GO:0006096~glycolytic process | 0.037352177 | 3.91 |
| GO_biological_processes | GO:0007626~locomotory behavior | 0.038188298 | 2.53 |
| GO_biological_processes | GO:0006935~chemotaxis | 0.040165559 | 2.18 |
| KEGG_pathway | hsa00591:Linoleic acid metabolism | 0.040543115 | 3.78 |
| GO_biological_processes | GO:0006641~triglyceride metabolic process | 0.040965189 | 3.80 |
| GO_biological_processes | GO:0006865~amino acid transport | 0.040965189 | 3.80 |
| GO_biological_processes | GO:0008203~cholesterol metabolic process | 0.041920546 | 2.74 |
| GO_biological_processes | GO:0009749~response to glucose | 0.041920546 | 2.74 |
| GO_biological_processes | GO:0019835~cytolysis | 0.042286818 | 5.07 |
| GO_biological_processes | GO:0016125~sterol metabolic process | 0.042286818 | 5.07 |
| GO_biological_processes | GO:0006544~glycine metabolic process | 0.042470726 | 8.87 |
| GO_biological_processes | GO:0007614~short-term memory | 0.042470726 | 8.87 |
| GO_biological_processes | GO:0072488~ammonium transmembrane transport | 0.042470726 | 8.87 |
| GO_biological_processes | GO:0090331~negative regulation of platelet aggregation | 0.042470726 | 8.87 |
| GO_biological_processes | GO:0070327~thyroid hormone transport | 0.042470726 | 8.87 |
| GO_biological_processes | GO:0045471~response to ethanol | 0.043775439 | 2.28 |
| GO_biological_processes | GO:0035690~cellular response to drug | 0.044520986 | 2.70 |
| GO_biological_processes | GO:0071333~cellular response to glucose stimulus | 0.044713088 | 3.07 |
| GO_biological_processes | GO:0009409~response to cold | 0.044769293 | 3.70 |
| GO_biological_processes | GO:0006956~complement activation | 0.044839931 | 2.45 |
| KEGG_pathway | hsa00140:Steroid hormone biosynthesis | 0.046805244 | 2.65 |
| KEGG_pathway | hsa00561:Glycerolipid metabolism | 0.046805244 | 2.65 |
| GO_biological_processes | GO:0006954~inflammatory response | 0.049012626 | 1.54 |
